# Targeted intracerebral delivery of the anti-inflammatory cytokine IL13 promotes alternative activation of both microglia and macrophages after stroke

**DOI:** 10.1186/s12974-018-1212-7

**Published:** 2018-06-04

**Authors:** Somayyeh Hamzei Taj, Debbie Le Blon, Chloé Hoornaert, Jasmijn Daans, Alessandra Quarta, Jelle Praet, Annemie Van der Linden, Peter Ponsaerts, Mathias Hoehn

**Affiliations:** 1 In-vivo-NMR Laboratory, Max Planck Institute for Metabolism Research, Gleuelerstrasse 50, D-50931 Köln, Germany; 2 Laboratory of Experimental Hematology, University of Antwerp, Antwerp, Belgium; 3 Vaccine and Infectious Disease Institute (Vaxinfectio), University of Antwerp, Antwerp, Belgium; 4 Bio-Imaging Laboratory, University of Antwerp, Antwerp, Belgium; 5 Department of Radiology, Leiden University Medical Center, Leiden, Netherlands

**Keywords:** Stroke, Microglia/macrophage polarization, Interleukin 13, Mesenchymal stem cells, Neuroinflammation

## Abstract

**Background:**

Subtle adjustment of the activation status of CNS resident microglia and peripheral macrophages, to promote their neuroprotective and neuroregenerative functions, may facilitate research towards curing neurodegenerative disorders. In the present study, we investigated whether targeted intracerebral delivery of the anti-inflammatory cytokine interleukin (IL)13, by means of transplanting IL13-expressing mesenchymal stem cells (IL13-MSCs), can promote a phenotypic switch in both microglia and macrophages during the pro-inflammatory phase in a mouse model of ischemic stroke.

**Methods:**

We used the CX_3_CR1^eGFP/+^ CCR2^RFP/+^ transgenic mouse model to separately recognize brain-resident microglia from infiltrated macrophages. Quantitative immunohistochemical analyses were applied to characterize polarization phenotypes of both cell types.

**Results:**

Distinct behaviors of both cell populations were noted dependent on the anatomical site of the lesion. Immunohistochemistry revealed that mice grafted with IL13-MSCs, in contrast to non-grafted and MSC-grafted control mice, were able to drive recruited microglia and macrophages into an alternative activation state, as visualized by a significant increase of Arg-1 and a noticeable decrease of MHC-II expression at day 14 after ischemic stroke. Interestingly, both Arg-1 and MHC-II were expressed more abundantly in macrophages than in microglia, further confirming the distinct behavior of both cell populations.

**Conclusions:**

The current data highlight the importance of controlled and localized delivery of the anti-inflammatory cytokine IL13 for modulation of both microglia and macrophage responses after ischemic stroke, thereby providing pre-clinical rationale for the application of L13-MSCs in future investigations of neurodegenerative disorders.

**Electronic supplementary material:**

The online version of this article (10.1186/s12974-018-1212-7) contains supplementary material, which is available to authorized users.

## Background

Ischemic stroke following cerebral artery occlusion is a major cause of chronic disability worldwide and an effective therapy to improve functional recovery after stroke is missing in the clinical settings [1]. A time-dependent recruitment and activation of immune cells, including brain-resident microglia, monocytes/macrophages, granulocytes, and T cells are a hallmark of the secondary damage after ischemia [2]. Under inflammatory condition caused by ischemia, there is a complex interaction between brain-resident microglia and infiltrated macrophages. These two phagocytic cell populations share many antigenic markers, although rapidly growing literature on this subject indicates that both cell types have distinct specialized functions [3, 4]. In this study, we performed stroke experiments in CX_3_CR1^eGFP/+^ CCR2^RFP/+^ knock-in fluorescent protein reporter mice, in order to distinguish brain-resident microglia from infiltrated macrophages after ischemic stroke. In these transgenic mice, the chemokine receptor CX_3_CR1 is mainly expressed by brain-resident microglia, while CCR2 is overexpressed in infiltrating activated macrophages [5]. A profound characterization of these cell populations in damaged CNS may provide the opportunity to manipulate specific subsets for therapeutic benefits.

In the early stage of ischemic stroke, these immune cells can acquire a protective function known as anti-inflammatory (M2) phenotype, i.e., presenting characteristic features comparable to in vitro IL4 and/or IL13 polarized cells. In the late stage of ischemic stroke, they can polarize into a classically activated state known as pro-inflammatory (M1) phenotype, i.e., presenting characteristic features comparable to in vitro LPS and/or interferon gamma (IFN-ϒ) activated cells [6]. Emerging knowledge in targeting cerebral inflammation argues that therapeutic approaches should move from extensive and forcible suppression of immune cells towards careful adjustment of the balance between their different phenotypes [7]. Therefore, to drive microglia and macrophages towards a more protective anti-inflammatory phenotype after stroke, we selected the immunomodulatory cytokine interleukin 13 (IL13), which is a well-known modulator of immune responses in vitro and in vivo [8, 9]. The neuroprotective importance of the cytokine IL13 has been demonstrated in several experimental models of neurodegenerative disorders. It can strongly decrease the pro-inflammatory cytokine secretion, reduce inflammatory cell infiltration, and suppress axonal loss [10, 11].

Effective delivery of therapeutic proteins, such as IL13 in the present study, is a main challenge in neuroinflammation research. To efficiently deliver IL13 to the ischemic brain, we used autologous mesenchymal stem/stromal cells (MSCs) genetically engineered to secrete IL13. As previously reported by us and others, MSCs can be considered as competent carrier cells, since they (i) are conveniently engineered with viral vectors to provide long-term gene expression [12, 13], (ii) have reduced immunogenicity because of a low MHC class I expression and the absence of MHC class II molecules and co-stimulatory factors [14], (iii) display a robust survival rate upon transplantation in CNS tissue [15, 16], (iv) have a potential immunomodulatory effect on natural killer cell function and B and T cell proliferation [17, 18], and (v) are able to produce immunomodulatory, neurotrophic, and angiogenic factors [19–21]. Therefore, the therapeutic relevance of MSCs in stroke models and other neurodegenerative disorders can be applied upon intracerebral or intraventricular administration.

The first aim of this study was to investigate the spatial distribution and the behavior of recruited resident microglia and infiltrating, peripheral macrophages in a transient focal cerebral ischemia model, to better understand the contribution of these cell populations to the inflammatory process after stroke. By using the CX_3_CR1^eGFP/+^ CCR2^RFP/+^ transgenic mouse model, our results have provided new insights into the trafficking and distinct functions of resident microglia and infiltrating macrophages in the post-ischemic brain. The second aim of this study was to discover whether MSC-based delivery of IL13 can modulate the spontaneous polarization shift of anti-inflammatory (M2) to pro-inflammatory (M1) phenotypes in microglia and macrophages during the second week after ischemic stroke. We agreed on applying the simplified classification of pro-inflammatory and anti-inflammatory states as M1 and M2 phenotypes, respectively, in order to best describe our intention of modulating the polarization of microglia and macrophages. Our results have provided confirmatory evidence that transplantation of IL13-MSCs, which continuously secrete IL13, is able to polarize both microglia and macrophages to a neuroprotective M2 phenotype during the pro-inflammatory status in ischemic stroke. These data strengthen the idea that IL13-MSCs could be considered as potent modulators of the cellular and molecular responses in neuroinflammation, with strong anti-inflammatory potential, for further exploration in animal models of stroke and other neurodegenerative disorders.

## Methods

### Animals and experimental groups

Wild type C57BL/6J mice were obtained via Charles River Laboratories (strain code 027) and were used for in vivo detection of IL13 mRNA produced by grafted IL13-MSCs. In total, 24 mice were included in the experiment, divided over the following three groups: (i) a non-injected control mice (*n* = 8), (ii) MSC-grafted mice (*n* = 8), and (iii) IL13-MSC-grafted mice (*n* = 8).

Transgenic CX_3_CR1^eGFP/eGFP^ mice (strain code 005582) and CCR2^RFP/RFP^ mice (strain code 017586) were obtained via Jackson Laboratories. CX_3_CR1^eGFP/+^ CCR2^RFP/+^ mice were obtained by breeding CX_3_CR1^eGFP/eGFP^ mice with CCR2^RFP/RFP^ mice. During the entire study, mice were kept in the animalarium of the University of Antwerp (UA) under normal day-night cycle (12/12) with free access to food and water. All animal experimental procedures were approved by the Ethics Committee for Animal Experiments of the UA (Approval No 2015-84 and 2018-36).

**Fig. 1:**
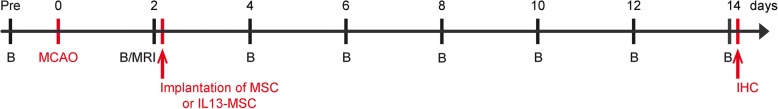
A time line of the experimental protocol. Stroke was induced in all mice via the middle cerebral artery occlusion (MCAO). All mice were scanned by MRI at day 2 after MCAO. MSCs or IL13-MSCs were transplanted into the right hemisphere, ipsilateral to the lesion, after performing MRI. Motor and sensory performance were tested and ranked according to the modified neurological deficit scores (mNDS), once before MCAO (baseline) and every 2 days after MCAO until the day of perfusion. All animals were sacrificed at day 14 after MCAO induction. Abbreviations: B = behavior, MCAO = middle cerebral artery occlusion, MRI = magnetic resonance imaging, IHC = immunohistochemistry

Ischemic stroke was induced in 32 male CX_3_CR1^eGFP/+^ CCR2^RFP/+^ mice (11–13 weeks, 22–26 g) by middle cerebral artery occlusion (MCAO), as described below. Stroke lesions were checked by magnetic resonance imaging (MRI) scans 48 h after MCAO, before transplantation of mesenchymal stem cells (MSCs). Two mice died after stroke induction (stroke-only group), one mouse before stroke during anesthesia. Four mice were excluded due to too small or absent stroke lesions. Eight mice died after cell implantation (*n* = 3 for MSC group; *n* = 5 for IL13-MSC group). In total, 17 mice were included in the study, resulting in the following three groups: (i) control group subjected to stroke only (*n* = 6), (ii) MSC-treated group, which received MSC transplantation 2 days after MCAO (*n* = 5), and (iii) IL13-MSC-treated group, which received a transplantation of IL13 producing MSCs 2 days after MCAO (*n* = 6). All mice were perfused at day 14 after stroke induction. An overview of the experimental protocol is presented in Fig. 1.

### Genetic engineering and culture of MSCs

In this study, we used a previously established and characterized C57BL/6 mouse bone marrow-derived MSC line [22] and a derivative thereof, genetically engineered to express murine interleukin-13 (further named as IL13-MSCs) [23]. The latter MSC line was generated by transduction of parental MSCs with the pCHMWS-IL13-IRES-Pac lentiviral vector, according to previously optimized procedures [24, 25]. For expansion, both MSC lines were cultured in standard cell culture plastic ware in “complete expansion medium” [24] consisting of Iscove’s modified Dulbecco’s medium (IMDM; Lonza) supplemented with 8% fetal bovine serum, 8% horse serum, 200 U/mL + 100 μg/mL penicillin/streptomycin, and 1 μg/mL amphotericin B (all products from Thermo Fisher Scientific). Culture medium for IL13-MSCs was further supplemented with 5 μg/mL puromycin (InvivoGen). MSC cultures were split 1:5 twice a week using 0.05% trypsin-EDTA (Thermo Fisher Scientific) for cell detachment.

### IL13 protein secretion assay

Wild type MSCs and IL13-MSCs were plated at a concentration of 2 × 10^5^/well in a six-well plate and allowed to adhere during overnight incubation. After the following 24 h of culture, supernatant was harvested and analyzed for the presence of IL13 protein by means of ELISA, according to the manufacturer’s instructions (Peprotech).

### Cell transplantation in healthy mouse brain

All surgical experiments were performed under sterile conditions, as previously described [26–28]. Briefly, mice were anesthetized by an intraperitoneal injection of a ketamine (80 mg/kg, Pfizer) + xylazine (16 mg/kg, Bayer Health Care) mixture in 0.9% NaCl solution (Baxter) and placed in a stereotactic frame (Stoelting). A midline scalp incision was made and a hole was drilled in the skull using a dental burr drill (Stoelting). Stereotactic coordinates were as follows (relative to bregma): AP 0 mm, Lat. 2.3 mm and − 2.3 mm, and DV − 2.3 mm. Next, an automatic microinjector pump (kdScientific) with a 10 μl Hamilton Syringe was positioned above the exposed *dura*. A 30-gauge needle (Hamilton), attached to the syringe, was lowered through the intact *dura* and positioned at the respective depth. After 2 min of tissue pressure equilibration, a suspension of 5 × 10^4^ MSCs in a volume of 0.4 μl was injected. Following 4 min to allow pressure equilibration and to prevent backflow of the injected cell suspension, the needle was fully retracted. The exact same procedure was performed at both left and right side of the brain. The skin was sutured (Vicryl, Ethicon), and 100 μl of a 0.9% NaCl solution was administered subcutaneously in order to prevent dehydration while mice were placed under a heating lamp to recover.

### qRT-PCR for transgenic IL13 mRNA

Mice were perfused with ice-cold 0.9% NaCl solution, directly followed by removal of the brain and dissection of the transplantation areas (left and right). The extracted tissue sections were snap-frozen in liquid nitrogen and kept at − 80 °C until further processing. As negative and positive control for qRT-PCR analysis, cultured MSCs and IL13-MSCs were harvested, washed, and resuspended in RNA*later* (Qiagen) solution at 4 °C for further processing the next day. Total RNA was extracted using the Purelink RNA kit (Invitrogen). RNA quantity and purity were determined using an ND-1000 micro-spectrophotometer (NanoDrop Technologies). Two micrograms of total RNA was reverse-transcribed using Omniscript RT kit (Qiagen). PCR primers were designed using online available primer design software from Thermo Fisher Scientific and were purchased from Thermo Fisher Scientific. The forward primer (5′ to 3′) GAAGCCGCTTGGAATAAGGC and the reverse primer (5′ to 3′) ACCTTGCATTCCTTTGGCGA cover part of the IRES sequence within the pCHMWS-IL13-IRES-Pac lentiviral construct, thereby allowing to detect only transgenic IL13 mRNA and not endogenous IL13 mRNA. Real-time quantitative RT-PCR analysis was carried out using Power SYBR Green PCR Master Mix (Applied Biosystems) detection, using the StepOnePlus Real-Time PCR System (Thermo Fisher Scientific). Thermal cycling conditions were 10 min at 95 °C and 40 cycles of 15 s at 95 °C and 1 min at 60 °C. Melt curves were performed upon completion of the cycles to ensure specificity of the product amplification. Housekeeping genes for normalization were peptidylprolyl isomerase A (ppiA) for detection of transgenic IL13 mRNA in control and MSC- and IL13-MSC-grafted mouse brains, using forward primer (5′ to 3′) CAGACGCCACTGTCGCTTT and reverse primer (5′ to 3′) TGTCTTTGGAACTTTGTCTGCAA and GAPDH for detection of transgenic IL13 mRNA in in vitro cultured MSC and IL13-MSC, using forward primer (5′ to 3′) AGGTCGGTGTGAACGGATTTG and reverse primer (5′ to 3′) GGGGTCGTTGATGGCAACA. Data were analyzed with qbase+ analysis software. For comparison purposes, obtained values for MSC-grafted and IL13-MSC-grafted mice are displayed as fold expression versus the mean value of control non-injected mice. Similarly, expression of transgenic IL13 mRNA for IL13-MSC is displayed as fold expression versus control MSC.

### Middle cerebral artery occlusion

Focal cerebral ischemia was induced by transient occlusion of the right middle cerebral artery (MCA) in all animals, using the intraluminal filament model as described previously [29, 30]. Briefly, mice were anesthetized with 1–2% isoflurane in a gas mixture of O_2_/N_2_O (30:70%) and received a subcutaneous injection of 4.0 mg/kg Carprofen (Pfizer, Karlsruhe, Germany) for analgesia after the surgical interventions. With a neck incision, the common carotid artery (CCA) and its proximal branches were exposed. The internal carotid artery (ICA) was occluded for a short time with a metal microvessel clip. A silicon rubber-coated filament with a tip diameter of 170 μm (7017PK5Re, Doccol Corporation, Sharon, MA, USA) was inserted into the ICA. The filament was advanced through the ICA until it blocked the blood flow to the middle cerebral artery. Animals were allowed to recover during the 30 min occlusion in a temperature stable box (MediHeat, Peco Services Ltd., Brough, UK). Afterwards, the animals were re-anesthetized, the filament was carefully removed to initiate reperfusion and the CCA was permanently ligated. During 1 week after the surgery, body weight was daily monitored in all animals. Animals were randomly assigned to the three different experimental groups.

### IL13-MSC injection following MCAO

All surgical experiments were performed as described earlier under section cell transplantation in healthy mouse brain. The needle was positioned at a depth of 2.5 mm into the striatum close to the ischemic lesion (as determined following MRI analysis). First, a suspension of 2 × 10^4^ MSCs in a volume of 0.4 μl was injected. Following 4 min of pressure equilibration, the needle was retracted to a depth of 1.0 mm and a second cell infusion (2 × 10^4^ MSCs in a volume of 0.4 μl) was performed.

### Behavioral tests

To observe the different aspects of neurological functions, a modified neurological deficit score (mNDS) was performed before and every 2 days after MCAO, based on a modification of a previous report [31]. In brief, the modified NDS consists of a set of motor tests (muscle status and abnormal movement), sensory tests (tactile, and proprioceptive), and reflex tests on a scale of 0–18. Increasing score indicates the severity of the stroke damage [32].

### MRI acquisition

At 2 days post induction of stroke, mice were subjected to MRI using a 7T Pharmascan MR scanner with a 16-cm-diameter horizontal bore (Bruker, Ettlingen, Germany). This system is equipped with a standard Bruker cross-coil setup, using a quadrature volume coil for excitation and an array mouse surface coil for signal detection. The system was interfaced to a Linux PC running Topspin 2.0 and Paravision 5.1 software (Bruker). Mice anesthesia was induced using 2% isoflurane (Forane Abbott, UK) in a gas mixture of 30% O_2_ and 70% N_2_ at a flow rate of 600 ml/min. During MRI acquisition, isoflurane concentration was set at 2%, and the respiration rate was continuously monitored using a pressure sensitive pad. In addition, body temperature was monitored via a rectal probe and was held constant between 37.0 and 37.3 °C using warm air coupled to a feedback unit (SA instruments, NY, USA). Both respiration and body temperature control systems were controlled by PC-Sam monitoring software (SA instruments, NY, USA). Following MR image acquisition, mice were left to recover separately under a heating lamp before returning to their respective cages. Following scout scans, we acquired an axial proton density weighted Rapid Acquisition and Relaxation Enhancement (RARE) image using the following parameters: repetition time (TR) = 3000 ms, echo time (TE) = 13.3 ms, matrix size (256 × 256), field of view (FOV) = (17.5 × 17.5) mm^2^, resolution = (0.068 × 0.068) mm^2^, 10 coronal slices, slice thickness = 0.8 mm, RARE factor = 8, and averages = 2. Next, for determination of the T2 values, we applied a multi-slice, multi-echo sequence based on the Carr-Purcell-Meiboom-Gill sequence. The following parameters were used: TR = 3000 ms, number of echoes = 10, echo spacing = 11 ms, matrix size (128 × 128), field of view (FOV) = (17.5 × 17.5) mm^2^, resolution = (0.137 × 0.137) mm^2^, 10 slices, and slice thickness = 0.8 mm.

### Immunofluorescent staining

All immunofluorescence analyses were performed according to previously described procedures [26, 28]. Briefly, mice were transcardially perfused with 0.9% NaCl solution followed by 4% paraformaldehyde (PFA). Next, brains were isolated and post-fixed with 4% PFA for 3 h, then dehydrated through a sucrose gradient of 5, 10, and 20%. Brains were snap-frozen in liquid nitrogen and kept at − 80 °C until further processing. Ten-micrometer-thick cryosections were made using a microm HM500 cryostat. Immunofluorescent staining was performed on brain slides using the following antibody combinations: a primary rabbit anti-RFP antibody (2.5 μg/ml, Abcam, ab62341) with a secondary donkey anti-rabbit Alexa Fluor 555 antibody (2 μg/ml, Thermo Fisher Scientific, A31572), a primary rat anti-F4/80 antibody (4 μg/ml, Bio-Rad, MCA497R) with a secondary goat anti-rat Cy5 antibody (10 μg/ml, Thermo Fisher Scientific, A10525), a primary rat anti-MHC-II antibody (2.5 μg/ml, eBioscience, 14–5321-82) with a secondary goat anti-rat Alexa Fluor 350 antibody (10 μg/ml, Thermo Fisher Scientific, A21093), and a primary goat anti-Arg1 antibody (4 μg/ml, Santa Cruz, sc-18354) with a secondary donkey anti-goat Alexa Fluor 350 (10 μg/ml, Thermo Fisher Scientific, A21081) and a primary rabbit anti-Ym1 antibody (1:50 dilution, StemCell Technologies 01404) with a secondary goat anti-rabbit Alexa Fluor 647 antibody (10 g/ml, Thermo Fisher Scientific, A21245). Before mounting with Prolong Gold antifade reagent, nuclear staining was performed using TOPRO-3 (1/200, Thermo Fisher Scientific). Images were acquired using a BX51 fluorescence microscope equipped with an Olympus DP71 digital camera. Olympus cellSens dimension software was used for image processing. In each brain section, five different regions of interest (ROIs) were selected with the constant exposure time: the border and the core region of the ischemic hemisphere in the striatum, the core region of the ischemic hemisphere in the cortex, and two ROIs in the cortex and striatum of the intact contralateral hemisphere.

### Quantitative immunohistochemical analysis

For quantitative phenotypic analysis of microglia and macrophage activities after stroke, immunofluorescence images were analyzed using NIH ImageJ analysis software (ImageJ) and TissueQuest 4.0 (TissueGnostics, Vienna, Austria). An entire picture of all ROIs, taken at × 20 magnification, was used for quantification. Cells were recognized based on the nuclei staining (nuclei size, staining intensity, and discrimination by area was optimized manually), followed by the analysis of specific staining. The cellular densities of eGFP^+^RFP^−^ microglia (CX_3_CR1^eGFP/+^), eGFP^−^RFP^+^ macrophages (CCR2^RFP/+^), and eGFP^+^RFP^+^ double-positive microglia/macrophages (CX_3_CR1^eGFP/+^ CCR2^RFP/+^) were quantified. To achieve optimal cell detection, the background threshold was defined. To discriminate false signals due to the coverage of CX_3_CR1^eGFP/+^ cell ramification with total nuclei intensity, the cut-off was defined for each ROI. Scattergrams were generated to visualize the corresponding positive cells in the source ROIs through the real-time back gating component. Mean intensity and the relative number of the co-expressed CX_3_CR1^eGFP/+^ or CCR2^RFP/+^ with pro- or anti-inflammatory markers F4/80, Arg-1, and MHC-II were obtained. Afterwards, the mean values were determined from analyses of at least three brain sections per mouse. Based on the above cell density analysis, the proportion of microglia or macrophages expressing F4/80, Arg-1, or MHC-II was calculated. To observe anti- and pro-inflammatory representative markers in double staining with eGFP^+^ or RFP^+^, the above-defined five different ROIs provided the following number of cells/mm^3^: (i) total cell density according to the nuclei staining of TOPRO-3, (ii) eGFP^+^ microglia cell density, (iii) RFP^+^ macrophage cell density, (iv) eGFP^+^RFP^+^ double-positive microglia/macrophages cell density, (v) F4/80^+^/eGFP^+^ or RFP^+^ cell density, (vi) Arg-1^+^/eGFP^+^ or RFP^+^ cell density, and (vii) MHC-II^+^/eGFP^+^ or RFP^+^ cell density.

### Statistics

For statistical analyses of qRT-PCR and ELISA data, GraphPad Prism software was used. For all other experiments, statistical analyses were performed with SPSS version 22 (IBMSPSS statistics, Ehningen, Germany). The Normality test and homogeneity of variances were assessed for all data. The nonparametric analysis approach, Kruskal-Wallis H, was performed for analysis of behavioral scores (mNDS). Immunohistochemistry (IHC) and qRT-PCR data from in vivo experiments were analyzed for significant changes between the three groups using one-way analysis of variance (ANOVA) with Bonferroni-corrected post hoc comparisons. qRT-PCR data and ELISA data for comparison of in vitro cultured MSCs and IL13-MSCs unpaired *t* tests were performed. Differences were considered statistically significant at a *p* value < 0.05. Data shown represent mean value per group ± standard deviation.

## Results

### In vivo transgenic IL13 mRNA production by grafted IL13-MSC

**Fig. 2:**
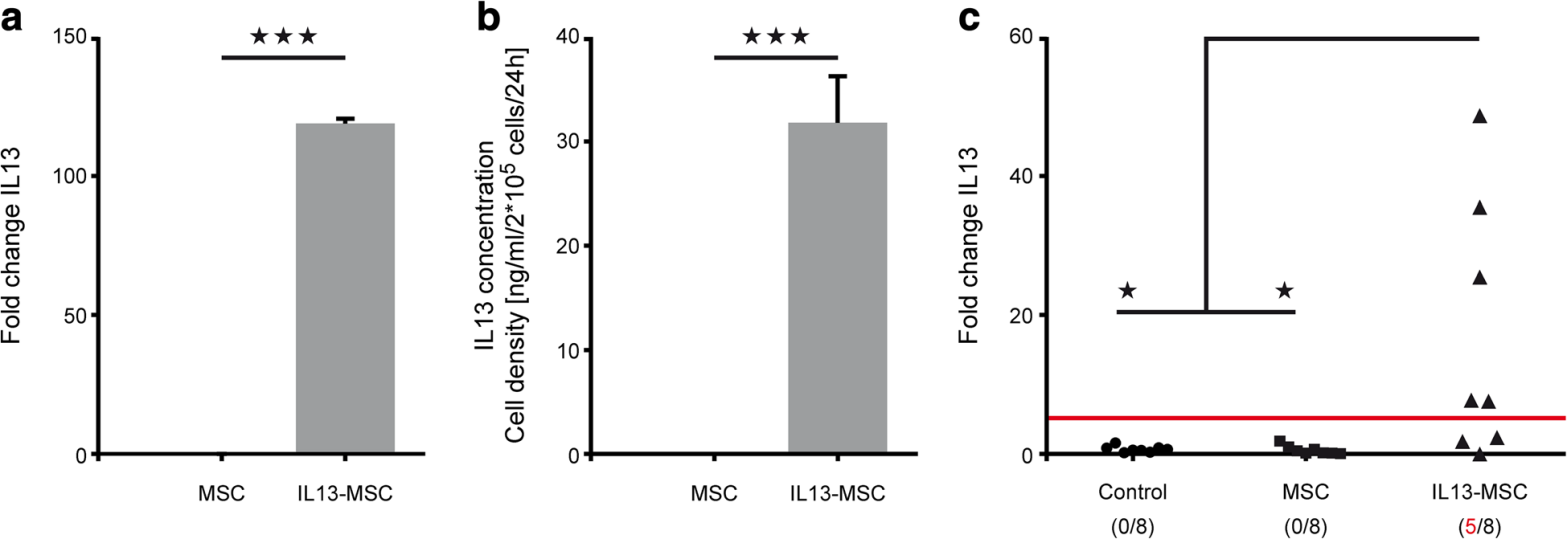
In vitro and in vivo expression of transgenic IL13 mRNA by IL13-MSCs. **a** Graph showing the analysis of transgenic IL13 mRNA expression by cultured MSCs and IL13-MSCs in vitro. **b** Graph showing the analysis of IL13 protein expression by cultured MSCs and IL13-MSCs after 24 h in vitro. **c** Graph showing the analysis of transgenic IL13 mRNA expression in the non-injected control group, the MSC-injected group, and the IL13-MSC-injected group at 1 week post-injection. Statistical significances are indicated by *** for *p* ≤ 0.001 and * for *p* ≤ 0.05

In our previous studies related to in vivo grafting of IL13-MSCs, we assumed transgenic IL13 mRNA (and protein) to be effectively produced as determined by the appearance of alternatively activated microglia and macrophages [23, 33, 34]. In this first part of our study, we aimed to determine whether IL13-MSCs effectively produce transgenic IL13 mRNA after in vivo transplantation. At first, results obtained from cultured MSCs and IL13-MSCs demonstrate that both mRNA and protein expression levels of IL13 are only detected in IL13-MSCs and not in MSCs (respectively; *p* < 0.0001 and *p* = 0.0003) (Fig. 2a, b). Next, following transplantation of MSCs and IL13-MSCs in healthy mouse brain, we were able to detect significant levels of transgenic IL13 mRNA expression in five out of eight IL13-MSC-injected mice at day 7 post-implantation, as compared to control non-injected (*p* = 0.0216) and MSC-injected mice (*p* = 0.0214) (Fig. 2c). Overall, these data demonstrate that IL13-MSC can effectively produce transgenic IL13 mRNA in situ following grafting in the CNS.

### Characterization of the ischemic lesion

**Fig. 3:**
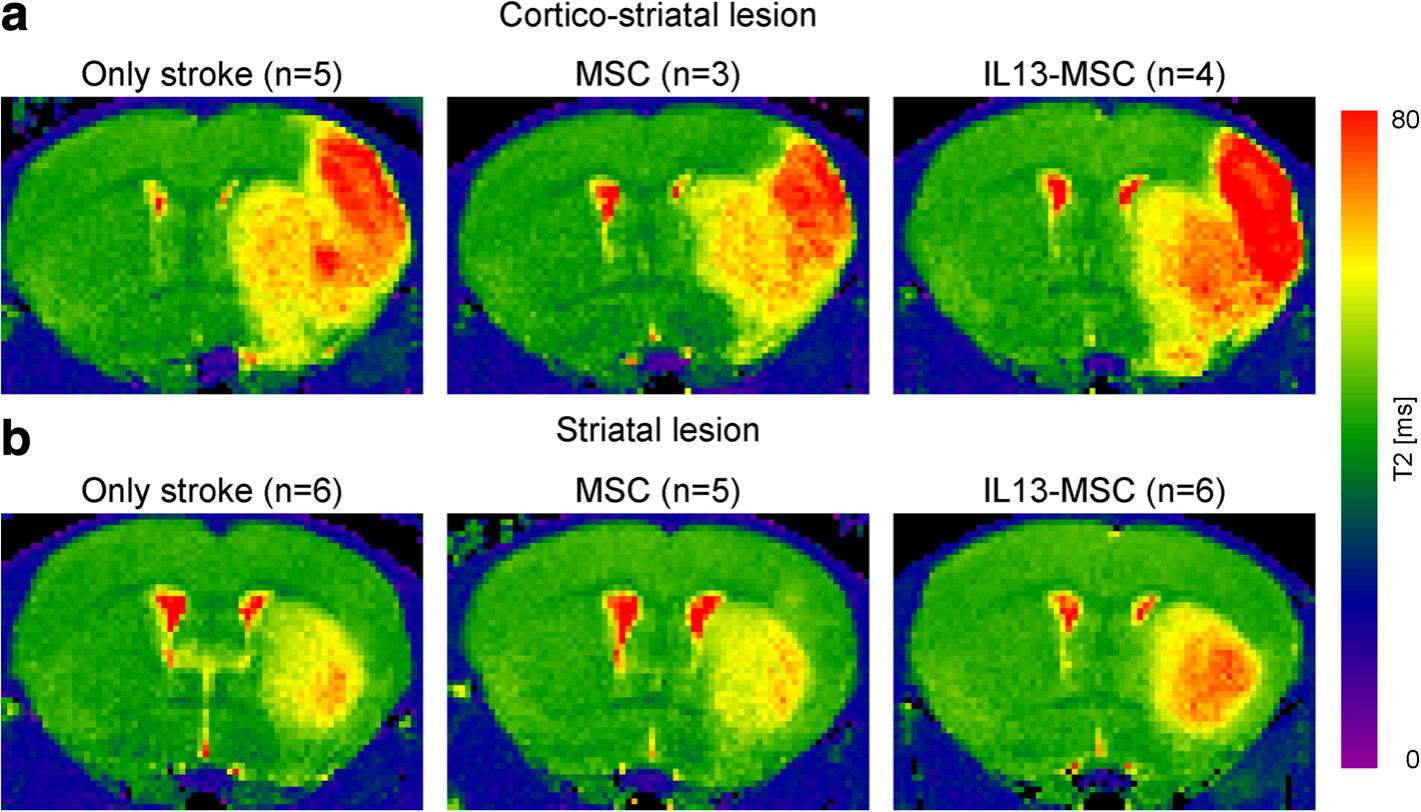
Representative T2 maps for lesion location and size of two representative animals from each experimental group 2 days after ischemic stroke. T2 maps are displayed as coronal brain section. Lesions show up as areas of elevated T2 values on T2 maps, representing the area with increased tissue water content following stroke induction. Two characteristic lesion types are visible in the right hemisphere 48 h after MCAO: **a** lesions involving both striatum and cortex (*n* = 12) and **b** lesions restricted to the striatum (*n* = 5)

Occlusion of the right middle cerebral artery with a silicone rubber-coated filament resulted in cerebral ischemia. Lesion size and location were assessed by MRI using quantitative T2 maps. Ischemic territory shows up as area of increased T2 values on T2 maps, representing the area with augmented tissue water content. In the hemisphere ipsilateral to the lesion, vasogenic edema increased T2 values and a clear lesion was detectable in the right ischemic hemisphere on T2 maps. In the intact contralateral hemisphere, T2 maps showed no signs of lesion. Two days after MCAO, two characteristic lesion types were detected: (1) in 5 mice, lesions were restricted to the striatum and (2) in 12 mice, lesions involved both striatum and cortex. Subsequently, mice were divided over three experimental groups: (1) control mice with untreated stroke, (2) mice with stroke receiving a control MSC graft, and (3) mice with stroke receiving an IL13-MSC graft. Representative lesions in each experimental group are displayed in Fig. 3.

### Neurological deficit

We determined whether the procedure of cell grafting (MSC or IL13-MSC) into the ischemic brain influences the motor and sensory performance after stroke in mice. In all three experimental groups, the neurological deficit scores increased at day 2 after MCAO (up to score 7 in mNDS) compared to baseline (score 0 in mNDS), indicating the impaired motor and sensory functions due to the stroke. No differences were observed between the three experimental groups within the two-week observation period. Nevertheless, our findings clearly indicate that the additional surgical procedure, intracranial MSC transplantation at 2 days after MCAO, did not worsen the neurological deficits after stroke (Additional file 1: Figure S1).

### Distinct spatial distribution of microglia and infiltrated macrophages following ischemic stroke

**Fig. 4:**
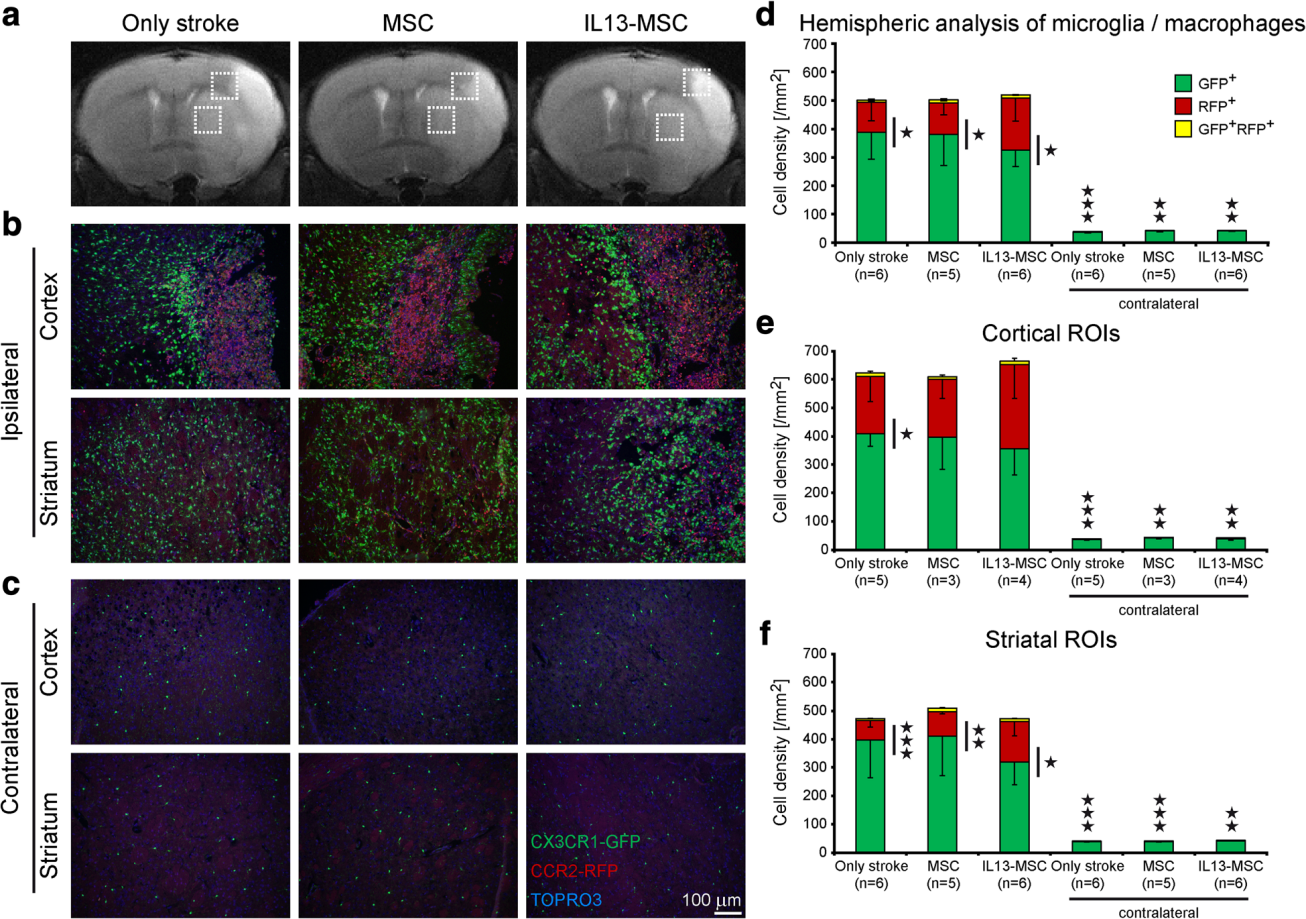
Localization and cell density of microglia and macrophages 2 weeks after ischemic stroke. **a** Representative coronal T2-weighted MR images are displayed above the corresponding immunohistochemistry images of all experimental groups. The hyperintense regions in the coronal MR images show the ischemic lesion 2 days post stroke. **b**–**c** Fluorescent microscopic images of brain coronal sections of all experimental groups represent the distribution of CX_3_CR1^eGFP^ microglia and CCR2^RFP^ macrophages at day 14 after stroke; **b** localization of eGFP^+^ microglia and RFP^+^ macrophages in ipsilateral cortical and striatal ischemic lesions, and **c** in contralateral cortical and striatal intact regions. Immunofluorescence colors: blue, TOPRO; green, microglia; red, macrophages. **d**–**f** Representative stacked column graphs show the cell density quantification of all experimental groups at day 14 after stroke. **d** Evaluation of microglia/macrophage cell density in the whole hemisphere. No significant difference was observed in the total number of eGFP^+^ microglia and RFP^+^ macrophages between the three experimental groups. CX_3_CR1^eGFP/+^ cell density is significantly higher than CCR2^RFP/+^ cell density in all three experimental groups at day 14 after stroke. In intact contralateral hemisphere, eGFP^+^ microglia was the main population. **e** Further evaluation of microglia and macrophage cell density in cortical lesion areas, and **f** in striatal lesion areas. The cell density of recruited microglia and macrophages was higher in large cortical lesion areas compared to striatal lesion areas. Data represent mean ± SD. The data were compared between the three experimental groups using a parametric one-way ANOVA test with Bonferroni’s post hoc test. To compare the CX_3_CR1^eGFP/+^ cell density to CCR2^RFP/+^ cell density in each group, an independent one-tailed Student’s *t* test was used

In order to histologically investigate brain trafficking of recruited microglia and macrophages after cerebral ischemia, we took advantage of CX_3_CR1^eGFP/+^ CCR2^RFP/+^ double transgenic mice. eGFP and RFP expression in the brain sections of these mice enables us to simultaneously track resident eGFP^+^ microglia (CX_3_CR1^eGFP/+^) and infiltrating RFP^+^ monocytes/macrophages (CCR2^RFP/+^). Using these transgenic mice, we observed differences in displacement of migration between microglia and peripheral monocyte-derived macrophages. Histological analysis revealed a noticeable infiltration of both microglia and macrophages throughout the ischemic territory at day 14 after stroke induction, (Fig. 4a–c). From the representative immunofluorescence images in Fig. 4b, we noted that eGFP and RFP expression shows a spatially different distribution in the cortex and the striatum. In the cortical part of the ischemic lesion, CCR2^RFP/+^ macrophages were located in the infarct core and CX_3_CR1^eGFP/+^ microglia were mainly accumulated at the infarct border. In contrast, in the striatal lesion area, CX_3_CR1^eGFP/+^ microglia and CCR2^RFP/+^ macrophages were diffusely distributed across the whole territory. The only population within the intact contralateral hemisphere contained resident CX_3_CR1^eGFP/+^ microglia (Fig. 4c). The microglia density on the ischemic hemisphere was significantly higher than on the contralateral hemisphere, in all groups and analysis (Fig. 4d–f). No obvious difference in this distribution pattern was observed between the three experimental groups.

Next, we assessed whether any differences in cell density existed between resident and infiltrating cells 2 weeks after stroke. When evaluating the whole lesion site (i.e., the cortical and/or striatal region), the vast majority of recruited cells within the lesion are CX_3_CR1^eGFP/+^ microglia in all three experimental groups. Statistical analysis confirmed that CX_3_CR1^eGFP/+^ cell density, being of microglial origin, is significantly higher than CCR2^RFP/+^ cell density, being of macrophage origin, in all three experimental groups at day 14 after stroke (stroke-only group *p* = 0.013, MSC-treated group *p* = 0.038, IL13-MSC-treated group *p* = 0.017) (Fig. 4d). When analyzing the cell density of microglia and macrophages, separately for striatal and for cortico-striatal lesions, the statistical significance between both cell populations was preserved in all cases, except for the two MSC groups in the cortical analysis (Fig. 4e), where only a trend was reached (MSC group *p* = 0.06; IL13-MSC group *p* = 0.063). Despite the difference in spatial distribution of microglia and macrophages between the striatal and cortical lesion area, further quantitative histological analysis revealed closely comparable numbers of infiltrating macrophages and of resident microglia between cortical (Fig. 4e) and striatal (Fig. 4f) lesions over the three experimental groups.

### Recruited CX_3_CR1^eGFP/+^ microglia exhibit various morphological phenotypes within the ischemic hemisphere

**Fig. 5:**
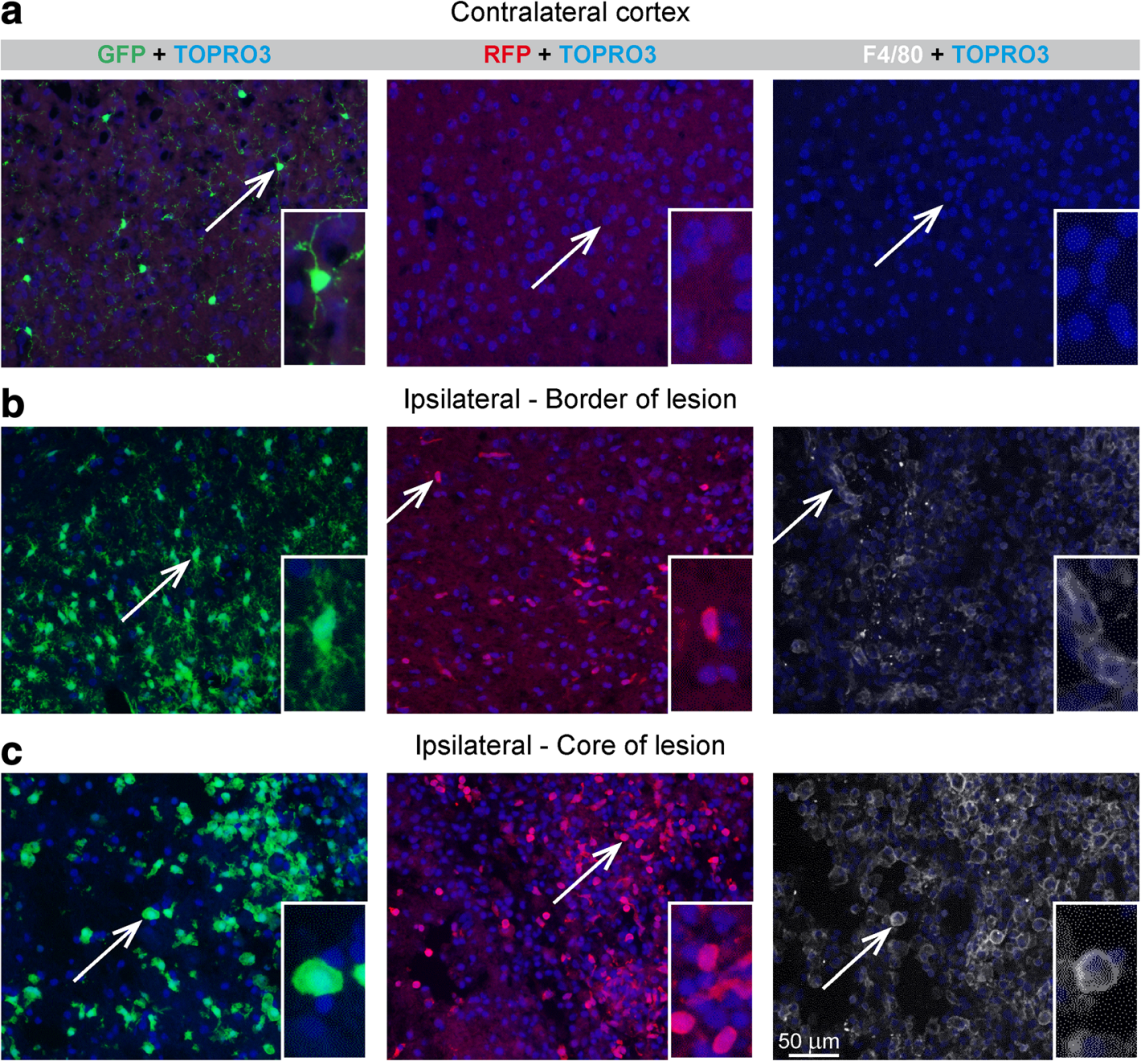
Different morphology of microglia and macrophages at day 14 after ischemic stroke. Representative photomicrographs with subsequent magnified images represent different morphology of microglia/macrophages in **a** intact contralateral hemisphere, **b** border of the ischemic lesion of ipsilateral hemisphere, and **c** the core of the ischemic lesion of ipsilateral hemisphere. eGFP^+^ microglia display at least three distinct phenotypes at day 14 after stroke including ramified cells (top row), intermediate cells (middle row), and amoeboid cells (bottom row). RFP^+^ macrophages show oval or round shape without apparent processes. F4/80^+^ cells, alternatively activated cells, display intermediate and amoeboid shape. Scale bar 50 μm

CX_3_CR1^eGFP/+^ microglia are visible in both the intact and the ischemic hemisphere, but are found in different numbers, distribution patterns (see above), and morphological appearance. In this study, we could observe at least three distinct morphological appearances of CX_3_CR1^eGFP/+^ microglia at day 14 after stroke induction (Fig. 5, left column): (i) cells with long thin arborized processes and a small cell body, defined as ramified cells (top row), (ii) cells with swollen processes and elongated amorphous larger cell body, classified as intermediate cells (middle row), and (iii) cells with round shape and no plasmalemmal processes, specified as amoeboid cells (bottom row). Ramified CX_3_CR1^eGFP/+^ cells were mostly observed in the healthy periphery of the lesion and in the contralateral hemisphere, but were not seen in the core of the lesion. Intermediate type CX_3_CR1^eGFP/+^ cells were closely associated with peri-infarct regions. Amoeboid CX_3_CR1^eGFP/+^ cells were mainly localized in the ischemic core. In contrast, recruited CCR2^RFP/+^ macrophages displayed a uniform oval, kidney form, or round shape without apparent processes (Fig. 5, central column). Some CCR2^+^ cells showed cellular processes, suggesting that these cells may acquire an intermediate phenotype in the damaged tissue. Examples in Fig. 5 were taken from the stroke-only group, but no obvious differences between the appearance of microglia and macrophage morphology was observed between the three experimental groups.

### Transplantation of IL13-producing MSCs increases the proportion of highly activated infiltrating macrophages

**Fig. 6:**
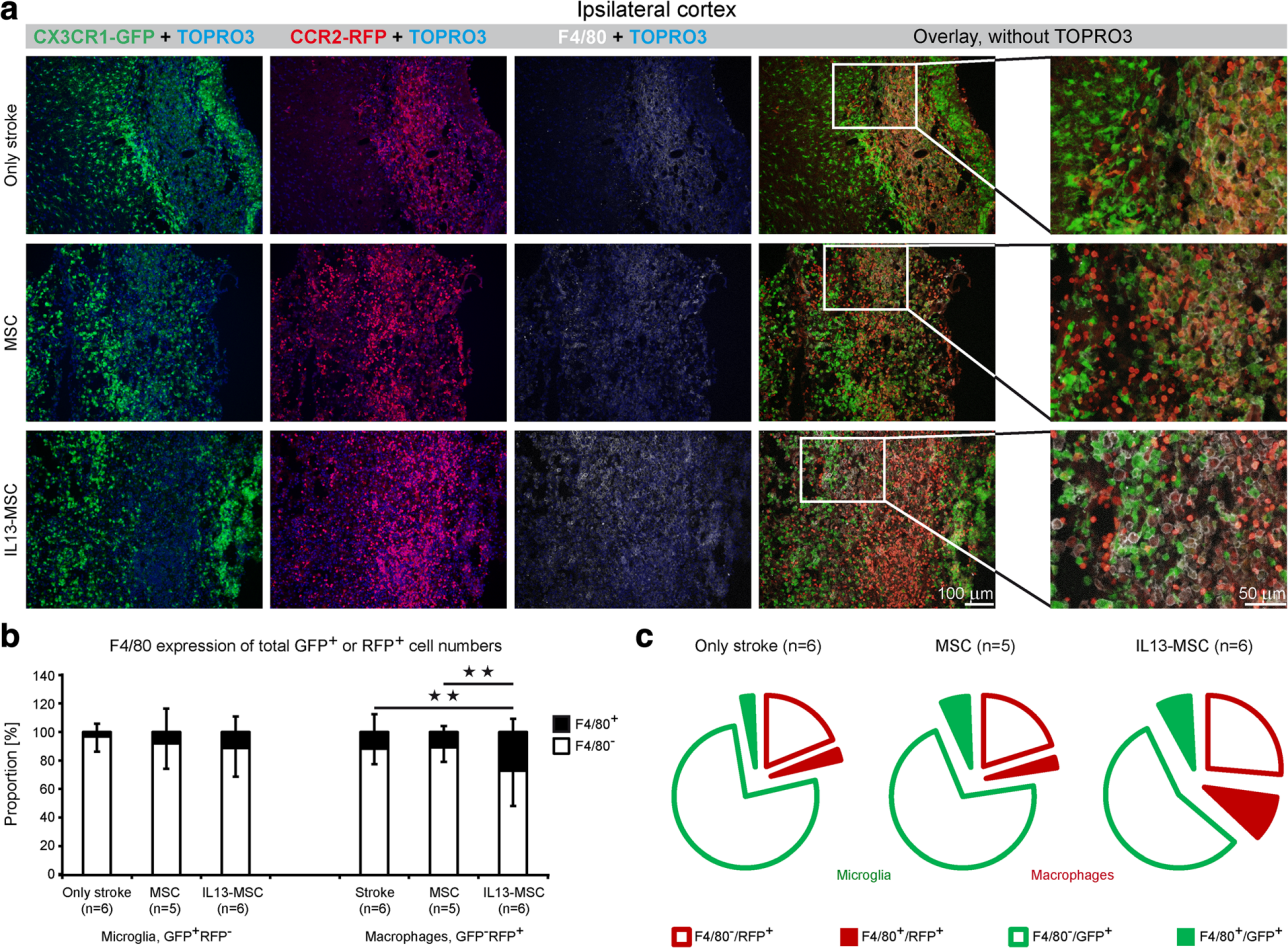
Transplantation of IL13-producing MSCs results in alternative activation of microglia and macrophages at day 14 after ischemic stroke. **a** Representative fluorescent microscopic images of ischemic cortical region display the expression of F4/80 on CX_3_CR1^eGFP/+^ and CCR2^RFP/+^ cells, acquired from the three experimental groups at day 14 after stroke. Illustrative ×20 close-up magnification represents the changes in F4/80 expression by microglia and macrophages. F4/80 biomarker is expressed on both cell populations in the hemisphere ipsilateral to the lesion. Immunofluorescence colors: blue, TOPRO; green, microglia; red, macrophages; white, F4/80. **b** Detailed analysis of the expression of biomarker F4/80 revealed a significant increase of F4/80 expression of CCR2^RFP/+^ macrophages upon IL13-MSCs transplantation in comparison to other two control groups. **c** The corresponding exploded pie charts show the distribution of F4/80 expression by GFP^+^ and RFP^+^ cells in all three experimental groups. In the pie chart, GFP^+^ microglia and RFP^+^ macrophages are encircled in green and red, respectively. *n* = 5–6 mice in each group. Data represent mean ± SD. The data were compared between the three experimental groups using a parametric one-way ANOVA test with Bonferroni’s post hoc test

Not only the recruitment of microglia and macrophages is a key feature of neuroinflammation, but also the subsequent phenotypic alterations associated with their activation status. Here, we first investigated the appearance of F4/80 expression, a general marker of activation, on microglia and macrophages following stroke (Fig. 5, right column). F4/80 expression was not detected contralateral to the lesion site (top row), while both microglia and macrophage populations (central and bottom row) within the lesion area, at least in part, express this F4/80 activation marker. Notably, morphological inspection of F4/80-expressing cells shows that these cells display an intermediate or amoeboid shape. Next, we assessed whether microglia and/or macrophage activation, based on F4/80 expression, was altered upon transplantation of IL13-producing MSC, as compared to the situation after transplantation of MSC alone or without any transplantation after stroke induction (Fig. 6). As shown by the representative immunofluorescent images in Fig. 6a, and further supported by the quantitative analysis provided in Fig. 6b (relative proportion of F4/80 expression, stacked bars) and Fig. 5c (absolute proportion of F4/80 expression, pie charts), F4/80 expression was observed on both microglia and macrophages in all three experimental groups. Interestingly, we observed a significantly increased expression of F4/80 by CCR2^RFP/+^ macrophages upon IL13-MSC transplantation as compared to F4/80 expression by CCR2^RFP/+^ macrophages following stroke or stroke + control MSC grafting (*p* = 0.008 and *p* = 0.007). The increased number of F4/80^+^ microglia detected following IL13-MSC grafting was not significantly different from other groups.

### Transplantation of IL13-producing MSC following stroke promotes the induction of alternatively activated microglia and macrophages

**Fig. 7:**
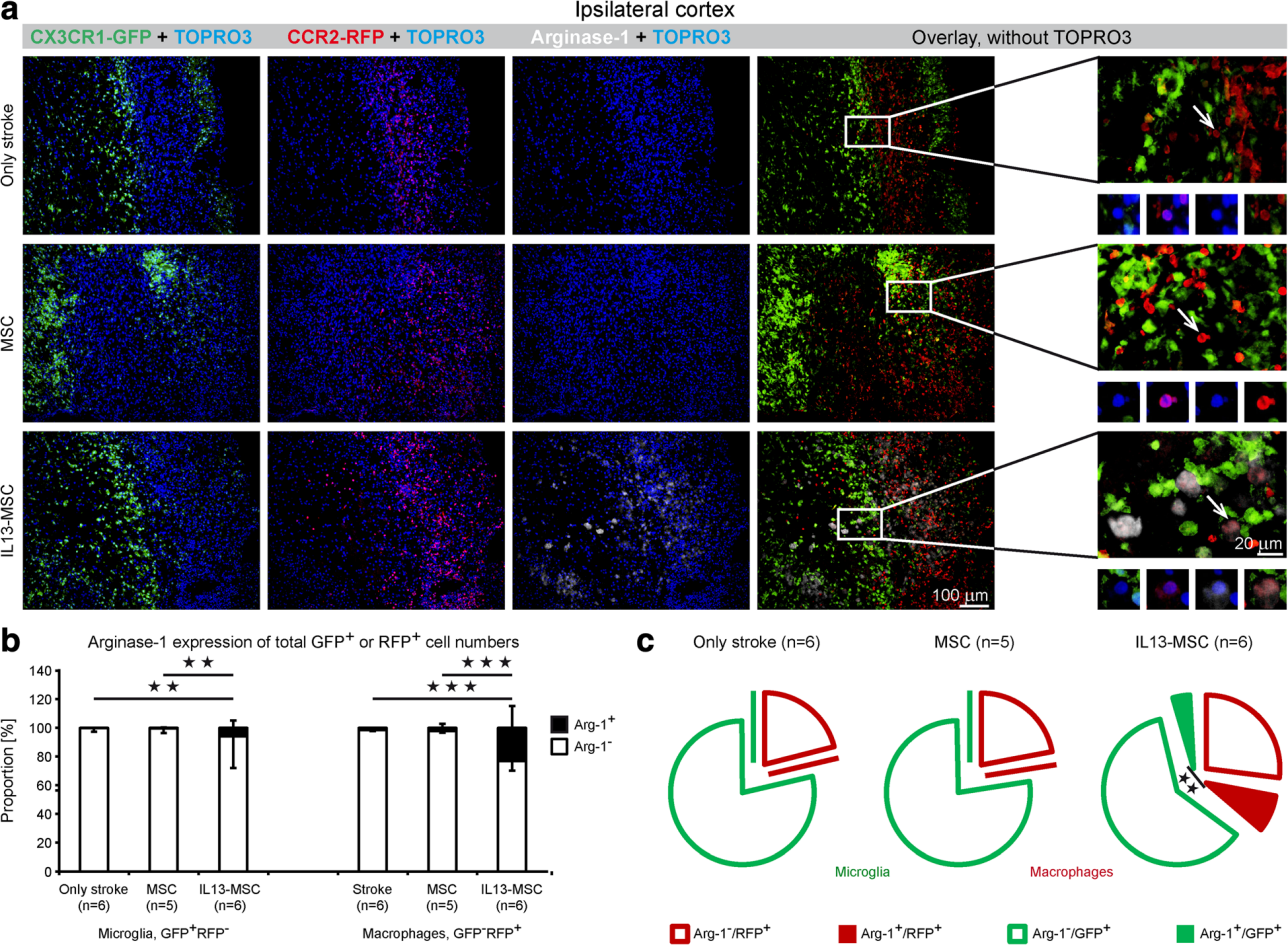
Transplantation of IL13-producing MSCs increases the number of recruited neuroprotective microglia and macrophages at day 14 after ischemic stroke. **a** Representative fluorescent microscopic images of ischemic cortical region show the expression of Arg-1 on CX_3_CR1^eGFP/+^ and CCR2^RFP/+^ cells in all three experimental groups at day 14 after stroke. Illustrative 20× close-up magnification display the changes in Arg-1 expression by microglia and macrophages. Immunofluorescence colors: blue, TOPRO; green, microglia; red, macrophages; white, Arg-1. **b** Detailed phenotypic quantitative analysis of CX_3_CR1^eGFP/+^ and CCR2^RFP/+^ cells expressing Arg-1 showed a significant increase in the number of GFP^+^RFP^−^Arg-1^+^ and GFP^−^RFP^+^Arg-1^+^ cells in IL13-MSC-treated group, in comparison to both control groups. **c** The corresponding exploded pie charts show the distribution of Arg-1 expression by GFP^+^ and RFP^+^ cells in all three experimental groups. In the pie chart, GFP^+^ microglia and RFP^+^ macrophages are encircled in green and red, respectively. In the IL13-MSC group, the Arg-1^+^ fraction of macrophages is significantly higher than that of microglia. *n* = 5–6 mice in each group. Data represent Mean ± SD. The data were compared between the three experimental groups using a parametric one-way ANOVA test with Bonferroni’s post hoc test

To further analyze the phenotype of recruited microglia and macrophages within the ischemic stroke lesion, as well as the effect of IL13-MSC thereon, we first performed additional immunofluorescence staining for Arg-1. Notably, we wanted to investigate whether IL13-producing MSC were able to convert stroke-associated neuroinflammatory immune responses into an alternatively activated inflammatory response. As shown by the representative immunofluorescent images in Fig. 7a, and further supported by the quantitative analysis provided in Fig. 7b (relative proportion of Arg1 expression, stacked bars) and Fig. 6c (absolute proportion of Arg1 expression, pie charts), significant increase in Arg1 expression was observed within and surrounding the lesion area on both microglia and macrophages following grafting of IL13-MSC (for microglia, stroke + IL13-MSC vs. stroke + MSC and stroke only, *p* = 0.002 and *p* = 0.005 respectively; for macrophages, stroke + IL13-MSC vs. stroke + MSC and stroke only, both *p* < 0.001). Furthermore, the induction of this alternative activation program, as characterized by Arg1 expression, was significantly higher in macrophages than in microglia in the IL13-MSC group (*p* = 0.002).

### Transplantation of IL13-producing MSC following stroke may reduce pro-inflammatory MHC-II expression on infiltrated macrophages

**Fig. 8:**
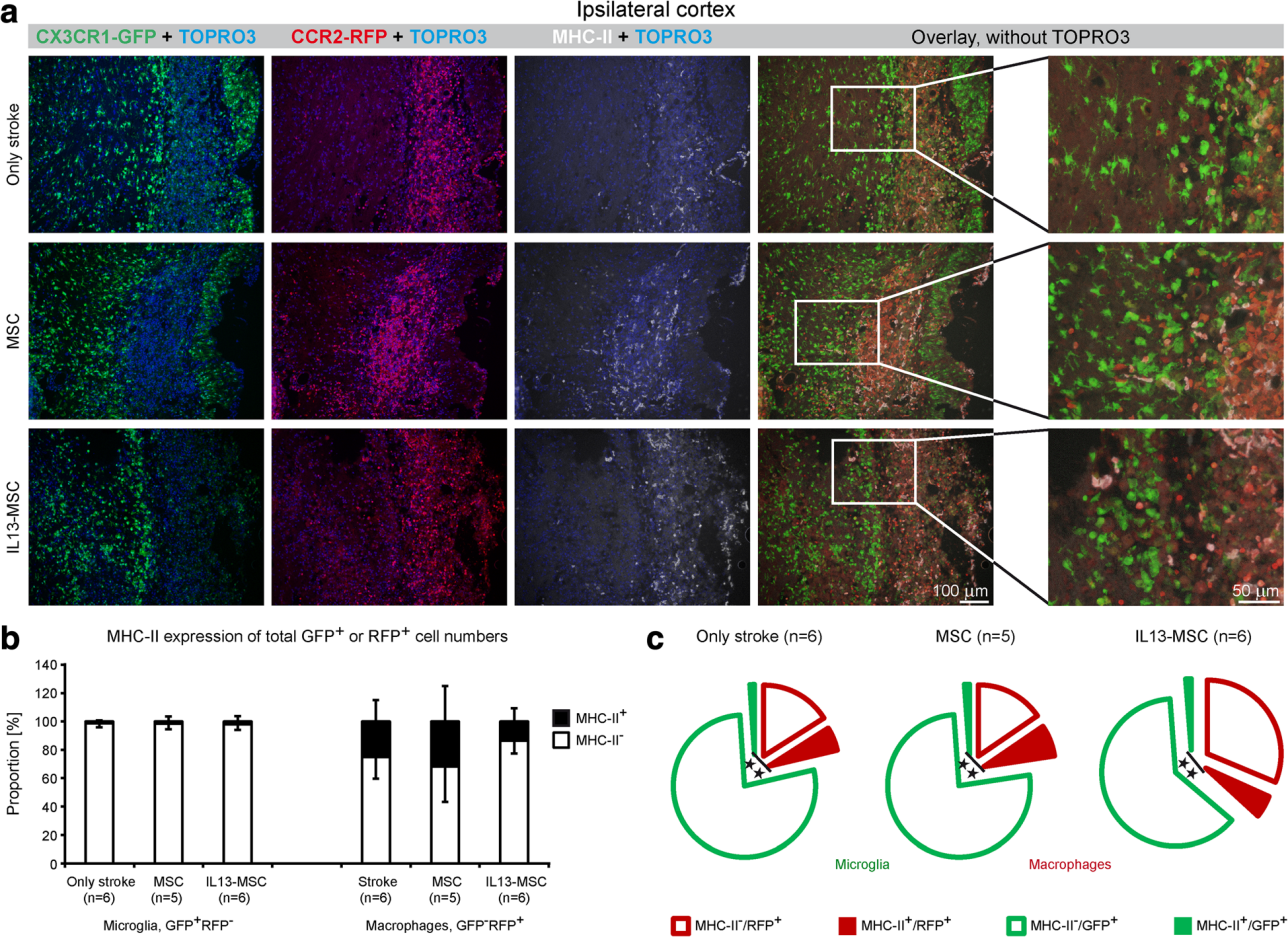
Transplantation of IL13-producing MSCs switches the polarization of microglia and macrophages towards less pro-inflammatory condition at day 14 after ischemic stroke. **a** Representative fluorescent microscopic images of ischemic cortical region show the expression of MHC-II on CX_3_CR1^eGFP/+^ and CCR2^RFP/+^ cells in all three experimental groups at day 14 after stroke. Illustrative ×20 close-up magnification displays the changes in MHC-II expression by microglia and macrophages. Immunofluorescence colors: blue, TOPRO; green, microglia; red, macrophages; white, MHC-II. **b** Detailed phenotypic quantitative analysis of CX_3_CR1^eGFP/+^ and CCR2^RFP/+^ cells expressing MHC-II showed a significant difference in the number of macrophages expressing MHCII, GFP^−^RFP^+^MHC-II^+^, in comparison to the number of microglia expressing MHC-II, GFP^+^RFP^−^MHC-II^+^, in all three experimental groups. In IL13-MSC-treated group, a trend towards less MHC-II expression by CCR2^RFP/+^ macrophages was detected, in comparison to both control groups. **c** The corresponding exploded pie charts show the distribution of MHC-II expression by GFP^+^ and RFP^+^ cells in all three experimental groups. In the pie chart, GFP^+^ microglia and RFP^+^ macrophages are encircled in green and red, respectively. In all three groups, the MHC-II^+^ fraction of macrophages is significantly higher than that of microglia. *n* = 5–6 mice in each group. Data represent mean ± SD. The data were compared between the three experimental groups using a parametric one-way ANOVA test with Bonferroni’s post hoc test

Likewise, we investigated the degree of MHC-II expression within and surrounding the lesion area over the different experimental conditions. As shown by the representative immunofluorescent images in Fig. 8a, MHC-II expression is observed mainly inside the ischemic lesion in all three experimental groups at day 14 after stroke induction. Further quantitative analyses provided in Fig. 8b (relative proportion of MHC-II expression, stacked bars) and 7C (absolute proportion of MHC-II expression, pie charts), indicated that MHC-II expression is significantly higher in CCR2^RFP/+^ macrophages, compared to CX_3_CR1^eGFP/+^ microglia (*p* = 0.008, *p* = 0.007 and p = 0.002, respectively, in each group). Following IL13-MSC grafting, no significant difference in MHC-II expression was observed on microglia. Even so, infiltrating macrophages seem to display less MHC-II expression following IL13-MSC grafting, albeit this decrease was not statistically significant.

### Characterization of MSC and IL13-MSC grafts

Similar to our preceding studies [23, 33–35], MSC and IL13-MSC grafts were able to survive in the pro-inflammatory stroke environment and displayed a similar remodeling pattern, MSC graft-infiltrating CCR2^RFP/+^ monocytes/macrophages at the core of the MSC grafts and brain-resident CX3CR1^eGFP/+^ microglia and astrocytes surrounding the MSC grafts . Expression of the two M2 markers Arginase1 and Ym1 by microglia and monocytes/macrophages, as a direct result of stimulation by IL13, was only detected in IL13-MSC grafts, but not in MSC grafts (Additional file 2: Figure S2).

## Discussion

The present study demonstrates that brain-resident microglia and infiltrated macrophages accumulate in the post-ischemic brain with distinct spatial patterns, indicating that they act as functionally different populations in CNS injury. While these two cell populations distinctly accumulate in the ischemic lesion in the cortex, they are randomly spread throughout the striatum. In addition to this region-dependent distribution, we here demonstrate a strong effect on modulation of the polarization of resident microglia and infiltrated macrophages through the IL13 secretion by transplanted MSCs after ischemic stroke. The locally secreted IL13 modulates the stroke-induced immune reaction by promoting an anti-inflammatory, M2-like phenotype of microglia and macrophages, as extensively visualized by arginase 1 expression, with the nature of the M2-like phenotype being further confirmed by the demonstration of Ym1 expression. This protective phenotype is much stronger in macrophages but also exists in microglia. The potent immunomodulatory effect of IL13 encourages to further evaluate the application of this cytokine in clinically relevant neurodegenerative disease models.

### Spatial distribution of microglia and infiltrated macrophages following ischemic stroke

The phenotypic distinction between CNS-infiltrating macrophages and brain-resident microglia is a major immunohistochemical concern. It is not only important in recognizing the origin of these cell populations, but it is also of fundamental importance in assessing the pathogenic and therapeutic significance of immune cells within the damaged brain. The lack of a single specific membranous and/or biochemical marker allowing definitive and discriminating identification of these cells is still a puzzling issue in neurobiology. Fortunately, alternative methods have been developed to overcome this hurdle, including the generation of bone marrow chimeric mice, which has proven to provide a powerful tool to distinguish microglia and infiltrated macrophages following ischemic stroke [36, 37]. In this study, however, we made use of the CX_3_CR1^+/GFP^CCR2^+/RFP^ transgenic mouse model, which is based on the fact that chemokine receptor CX_3_CR1 is predominantly expressed by CNS resident microglia and that CCR2 is upregulated in activated infiltrated macrophages and therefore allows distinction of eGFP-expressing microglia from RFP-expressing infiltrated macrophages [5]. Through the use of this transgenic mouse model and immunofluorescence microscopy, we were able to demonstrate a region-dependent distribution of microglia and macrophages at day 14 after stroke. In the cortical ischemic lesion, CX_3_CR1^+^ microglia distinctly accumulated at the border of the lesion, while CCR2^+^ macrophages were localized at the core of the lesion. At day 14, after ischemic stroke, CX_3_CR1^+/GFP^ microglia were the major contributors of recruited cells to the ischemic lesion. These observations are in line with our earlier findings following MSC transplantation in mouse brain, which demonstrated a similar distinct distribution of microglia and macrophages, where infiltrated macrophages invaded the hypoxic/ischemic MSC transplant core, while active microglia remained in the surrounding border [23, 25, 38]. In contrast to their orderly distribution in the cortex, both CX_3_CR1^+^ microglia and CCR2^+^ macrophages were found to be randomly distributed in striatal lesions. In agreement with our own observations, others have also reported that resident microglia and blood-derived macrophages localize in the ischemic brain with different temporal and spatial patterns. Garcia-Bonilla et al. describe that at the first week after stroke, mostly diffused CCR2^+^ macrophages were observed throughout the ischemic lesion, while during the second and third week after stroke, they saw that CX_3_CR1^+^ cells were localized in the peri-infarct area encircling the ischemic lesion core filled with CCR2^+^ macrophages [39]. Indeed, in a rather complex study design, these latter authors demonstrated that CCR2^+^CX3CR1^−^ monocytes may, in a time-dependent fashion after infiltration, turn into CCR2^−^CX3CR1^+^ macrophages. Thus, we must caution that our assignment of green fluorescent, CX3CR1-positive microglia may to some extent also contain macrophages. This, however, does not affect our assignment of CCR2^+^ red cells as a pure macrophage population. Another study showed that infiltrated blood-borne monocytes were exclusively located at the ischemically injured striatum at days 3, 7, and 14 after MCAO [40]. Their results represent a random distribution of brain-resident microglia and infiltrated monocyte within the striatum after ischemic stroke, with microglia being the vast majority of cells invading the ischemic lesion. Moreover, in a mouse model of traumatic spinal cord injury, CX_3_CR1^+/GFP^ and CCR2^+/RFP^ cells were randomly distributed around and inside the lesion, with CCR2^+/RFP^ cells constituting the greater number of accumulated cells in the lesion area [41].

These findings suggest an unknown mechanism by which temporally and spatially distinct ischemic regions can differentially signal to microglia and macrophages. Region-specific differences between cortex and striatum in regard to vascular volume [30], neurogenic potential [42] and neuroinflammatory responses [43] may cause the region-dependent distribution of microglia and macrophages after ischemic stroke.

In our experimental setup, considering CX_3_CR1^+/GFP^ cells as brain-resident microglia, it might be argued that perivascular macrophages, supraependymal macrophages, epiplexus cells of the choroid plexus, and meningeal macrophages express CX_3_CR1 too [44]. In addition, we cannot neglect the presence of patrolling macrophages Ly6c^−^ CX_3_CR1^high^ CCR2^low^ in the ischemic brain with disrupted blood-brain barrier [41, 45]. Interestingly, in an attempt to observe chemokine receptor expression changes in microglia and monocytes/macrophages in development and during inflammatory condition, Mizutani and colleagues have indicated that CCR2 is absent in adult naïve and inflamed CNS resident microglia [5]. Importantly, they have demonstrated that CX_3_CR1 expression by microglia is significantly higher compared to CX_3_CR1^low^ CCR2^high^ or CX_3_CR1^high^ CCR2^low^ monocytes/macrophages. Therefore, we hypothesize that CX3CR1^+/GFP^ cells are mostly of brain-resident microglial origin and double-positive cells for CX_3_CR1^+/GFP^ and CCR2^+/RFP^ are blood-derived macrophage populations [33, 41, 46]. It should be emphasized that the presence of double-positive cells at day 14 after ischemic stroke accounts for a very small proportion of infiltrated macrophages in our experiment. Further assessment of earlier or later time point of macrophage recruitment following CNS damage should also be evaluated. Nevertheless, the discovery of more microglia-specific markers would contribute to more efficient discrimination between CNS resident microglia and infiltrated macrophages in future studies.

### Dynamic modulation of microglia and infiltrated macrophages following ischemic stroke

In this study, we are the first to determine the immunomodulatory potential of MSC and IL13-expressing MSC engraftment in adjusting the polarization of brain-resident microglia and infiltrated macrophages in an ischemic stroke model. We focused on addressing important applications of IL13-expressing MSCs as a new potential therapeutic approach for ischemic stroke. The main goal of our study was to enhance the therapeutic effects of MSC transplantation following ischemic stroke by boosting the immunomodulatory properties of MSCs. For this reason, we used MSCs as a biodegradable delivery system for the immune modulating cytokine IL13. With this method, we achieved long-term effect on immune function until the second week after stroke induction, during the pro-inflammatory peak of stroke [6]. Earlier investigations by us, on the use of MSCs as a delivery system for the anti-inflammatory cytokine IL13, demonstrated that continued secretion of IL13 by MSCs is able to limit microgliosis, oligodendrocyte loss, and demyelination in cuprizone-treated mice, a model for neuroinflammation and demyelination [23], and promotes histopathological and functional recovery following spinal cord injury in mice [33]. In these studies, we also showed that transplantation of IL13-expressing MSCs induces alternative activation in both MSC graft-invading macrophages and in MSC graft-surrounding microglia, characterized by the expression of Arg-1 and Ym1 as anti-inflammatory-associated markers.

Here, we present for the first time that transplantation of IL13-expressing MSCs at 48 h after ischemia shifts major players of the immune reaction, namely microglia and macrophages, towards an anti-inflammatory, neuroprotective phenotype at 14 days after ischemia. Although not investigated in detail in this study, our previous data with regard to grafting IL-13 expressing MSCs in muscle of brain tissue have shown that the anti-inflammatory M2 phenotype can be induced already at 5 and 7 days post grafting, respectively [35]. The transplantation of IL13-expressing MSCs at day 2 after ischemia caused a noticeable increase in the expression of the general activation marker F4/80, especially by infiltrated macrophages. Most importantly, MSC graft-associated microglia and infiltrated macrophages were strongly driven towards an Arg-1^+^ alternative activation status at day 14 after stroke. Further distinction between recruited brain-resident microglia and infiltrated systemic macrophages following stroke and MSC transplantation in CX_3_CR1^eGFP/+^ CCR2^RFP/+^ double transgenic mice revealed that about two thirds of Arg-1^+^ cells are infiltrated macrophages. Evidently, continued secretion of the cytokine IL13 by MSCs was able to boost the immunomodulatory properties of MSCs by increasing the number of Arg-1^+^ cells, thus acting as a potent neuroprotective mediator [47]. Several groups have reported that Arg-1 expression by different cell types, including astrocytes, neurons, and immune cells, notably decreases during the first few days after cerebral ischemia, after an early peak at day 3 [6, 48]. By continuous secretion of IL-13 via MSCs, we could enhance and prolong the expression of neuroprotective and anti-inflammatory marker Arg-1 until week 2 after stroke induction. In an earlier study by our laboratory, in which we attempted to modulate the polarization of microglia and macrophages by microRNA-124 after ischemic stroke, we achieved an upregulation of Arg-1 and CD-206 expression by microglia and macrophages until day 6 after stroke. In this study, the increased polarization of microglia and macrophages towards the more anti-inflammatory phenotype was positively correlated to increased neuronal survival and functional recovery at day 6 after stroke [32, 49].

Another important observation to be taken into account here is the expression and accumulation of major histocompatibility complex class II (MHC-II) following MSC and IL13-expressing MSC engraftment after stroke. MHC-II expression was predominantly observed on infiltrated macrophages, localized at the core of the ischemic lesion. MHC-II is considered to be a pro-inflammatory mediator, which is overexpressed under a wide range of pathological conditions [50]. However, some studies have highlighted that MHC-II expression can also provide protective and neurotrophic functions and is involved in regeneration and axon integrity in neurodegenerative disorders [51, 52]. As reported previously, accumulation of CD11b^+^ MHC-II^+^ cells in the ipsilateral ischemic hemisphere after transient MCAO provides trophic support and is effective in the remyelination process after stroke [53]. With regard to the expression of Arg-1 and MHC-II, mostly by macrophages at the core of ischemic lesion in our study, we could argue a substantial role of infiltrated macrophages for the regeneration and repair process of injured brain. We propose that infiltrated pro-inflammatory CCR2^+^MHC-II^+^ macrophages may exert some pro-neurogenic functions such as clearance of cell debris after brain injury, which could be efficient at neurogenesis and regeneration process after ischemic stroke. Based on our data, transplantation of IL13-expressing MSCs does not broadly suppress M1 phenotype of microglia and macrophages, but it rather adjusts the balance between pro- and anti-inflammatory immune cell processes. As M1-activated cells typically resolve injured and/or infected tissue, which can lead to detrimental exacerbated injury of healthy tissue, they can also assist in the repair/conservation of synaptic connectivity and axonal regeneration [54, 55]. When considering M2 activated immune cells, mainly anti-inflammatory and regeneration-inducing functions are described, but it is worth noting that long-term maintenance of anti-inflammatory phenotype may adversely affect the immune response, which leads to serious negative effects, such as tumorigenesis [56]. Induction of an M1/M2 phenotype switch to improve natural repair processes in neurodegenerative disorders should be considered with caution. In stroke, our earlier studies on modulation of the pro-/anti-inflammatory balance by microRNA-124 demonstrated enhanced neuronal survival and behavioral improvement [32, 49].

In our present investigation, we have not analyzed the influence of continuous secretion of the anti-inflammatory cytokine IL-13 and/or M2 polarized microglia and macrophages on the survival of autologous MSC grafts after stroke. But in an earlier study, we have demonstrated that transplantation of IL13-expressing MSCs leads to a notable decrease of direct and indirect immune recognition and rejection of cell grafts [35]. Further support of functional interaction between alternative activation status of surrounding inflammatory milieu at the graft site and survival of MSCs comes from a study by Yu et al. They have recently demonstrated that M2 macrophage-secreted OA/GPNMB, osteoactivin/glycoprotein in non-metastatic melanoma protein B, positively contributes to viability, proliferation, and migration of MSCs, through ERK and AKT signaling pathways [57]. Similar MSC survival improvement was achieved via an ischemic microenvironment in vitro study, in which macrophage migration inhibitory factor (MIF), a cytokine expressed by activated monocytes/macrophages, protects MSCs from apoptosis via a CD74-dependent Akt-FOXO3a-related pathway [58]. Enhancing MSC survival in the face of oxygen and nutrient deprivation that naturally occur in the ischemic stroke and cell graft procedure clearly needs further investigation.

## Conclusions

Our findings will serve as a base for future studies to apply MSCs in a more effective way to improve CNS repair. We have identified that MSCs expressing the anti-inflammatory cytokine IL13 serve as an excellent candidate for more effective modulation of inflammatory responses in neurodegenerative disorders. This new insight could have noticeable therapeutic implications, which demands further studies using human biological systems, such as human-derived mesenchymal stem cells, for translation of immune response modulation research into clinical practice.

## Additional files

Additional file 1: **Figure S1.** Motor and sensory performance after ischemic stroke. Behavioral performance was assessed by the modified neurological deficit scores (mNDS), before and every 2 days after MCAO. In all three groups, the mNDS increased significantly at day 2 after MCAO compared to baseline. Graphs of all three experimental groups show no significant difference among the groups. The procedure of cell grafting into the ischemic brain and the additional surgical procedure did not worsen the motor and sensory performance after stroke. *n* = 5–6 mice in each group. Data are mean ± SD. The data were compared between the three experimental groups using the nonparametric Kruskal-Wallis H test. (TIF 837 kb)

Additional file 2: **Figure S2.** Representative example of MSC and IL13-MSC graft site remodeling within the MCAO brain lesion site. Control MSC (upper panel) and IL13-MSC (lower panel) grafts are able to survive in the pro-inflammatory stroke environment and display a similar remodeling pattern. MSC graft-infiltrating CCR2^RFP/+^ monocytes/macrophages (in red) at the core of the MSC grafts and brain-resident CX3CR1^eGFP/+^ microglia (in green) and astrocytes (in blue, first row) surrounding the MSC grafts. Arginase1 expression (in blue, second and third row) and Ym1 expression (in magenta, third row) by microglia and monocytes/macrophages, as a direct result of stimulation by IL13, was only detected in IL13-MSC grafts, but not in control MSC grafts. Scale bar 100 μm. (TIF 13199 kb)

## Abbreviations

AKT: Alpha serine/threonine-protein kinase
Arg-1: Arginase-1
CCA: Common carotid artery
CCR2: CC-chemokine receptor 2
CD11b: Clusters of differentiation 11b
CNS: Central nervous system
CX_3_CR1: CX_3_C chemokine receptor 1
EAE: Experimental autoimmune encephalomyelitis
ERK: Extracellular signal-regulated kinases
FOXO3: Forkhead box O3
GAPDH: Glyceraldehyde-3-phosphate dehydrogenase
Iba-1: Ionized calcium-binding adapter molecule 1
IFN-ϒ: Interferon gamma
IHC: Immunohistochemistry
IL: Interleukin
IL13-MSC: IL13-producing MSCs
LPS: Lipopolysaccharide
MCA: Middle cerebral artery
MCAO: Middle cerebral artery occlusion
MHC-II: Major histocompatibility complex class II
MIF: Macrophage migration inhibitory factor
miR-124: MicroRNA-124
mNDS: Modified neurological deficit score
MRI: Magnetic resonance imaging
MSCs: Mesenchymal stem cells
PBS: Phosphate-buffered saline
PFA: Paraformaldehyde
ppiA: Peptidylprolyl isomerase A
RT: Room temperature

## References

[1] Donnan GA, Fisher M, Macleod M, Davis SM (2008). Stroke Lancet.

[2] Jin R, Yang G, Li G (2010). Inflammatory mechanisms in ischemic stroke: role of inflammatory cells. J Leukoc Biol.

[3] Patel AR, Ritzel R, McCullough LD, Liu F (2013). Microglia and ischemic stroke: a double-edged sword. Int J Physiol Pathophysiol Pharmacol.

[4] Shechter R, Schwartz M (2013). Harnessing monocyte-derived macrophages to control central nervous system pathologies: no longer ‘if’ but ‘how’. J Pathol.

[5] Mizutani M, Pino PA, Saederup N, Charo IF, Ransohoff RM, Cardona AE (2012). The fractalkine receptor but not CCR2 is present on microglia from embryonic development throughout adulthood. J Immunol.

[6] Hu X, Li P, Guo Y, Wang H, Leak RK, Chen S (2012). Microglia/macrophage polarization dynamics reveal novel mechanism of injury expansion after focal cerebral ischemia. Stroke.

[7] Hu X, Leak RK, Shi Y, Suenaga J, Gao Y, Zheng P (2015). Microglial and macrophage polarization-new prospects for brain repair. Nat Rev Neurol.

[8] Doherty TM, Kastelein R, Menon S, Andrade S, Coffman RL (1993). Modulation of murine macrophage function by IL-13. J Immunol.

[9] Doyle AG, Herbein G, Montaner LJ, Minty AJ, Caput D, Ferrara P (1994). Interleukin-13 alters the activation state of murine macrophages in vitro: comparison with interleukin-4 and interferon-gamma. Eur J Immunol.

[10] Offner H, Subramanian S, Wang C, Afentoulis M, Vandenbark AA, Huan J (2005). Treatment of passive experimental autoimmune encephalomyelitis in SJL mice with a recombinant TCR ligand induces IL-13 and prevents axonal injury. J Immunol.

[11] Ochoa-Reparaz J, Rynda A, Ascon MA, Yang X, Kochetkova I, Riccardi C (2008). IL-13 production by regulatory T cells protects against experimental autoimmune encephalomyelitis independently of autoantigen. J Immunol.

[12] Lee K, Majumdar MK, Buyaner D, Hendricks JK, Pittenger MF, Mosca JD (2001). Human mesenchymal stem cells maintain transgene expression during expansion and differentiation. Mol Ther.

[13] Ozawa K, Sato K, Oh I, Ozaki K, Uchibori R, Obara Y (2008). Cell and gene therapy using mesenchymal stem cells (MSCs). J Autoimmun.

[14] Ankrum JA, Ong JF, Karp JM (2014). Mesenchymal stem cells: immune evasive, not immune priviledged. Nat Biotechnol.

[15] De Vocht N, Lin D, Praet J, Hoornaert C, Reekmans K, Le Blon D (2013). Quantitative and phenotypic analysis of mesenchymal stromal cell graft survival and recognition by microglia and astrocytes in mouse brain. Immunobiology.

[16] Praet J, Reekmans K, Lin D, De Vocht N, Bergwerf I, Tambuyzer B (2012). Cell type-associated differences in migration, survival, and immunogenicity following grafting in CNS tissue. Cell Transplant.

[17] Le Blanc K (2003). Immunomodulatory effects of fetal and adult mesenchymal stem cells. Cytotherapy.

[18] Krampera M, Glennie S, Dyson J, Scott D, Laylor R, Simpson E (2003). Bone marrow mesenchymal stem cells inhibit the response of naive and memory antigen-specific T cells to their cognate peptide. Blood.

[19] Quertainmont R, Cantinieaux D, Botman O, Sid S, Schoenen J, Franzen R (2012). Mesenchymal stem cell graft improves recovery after spinal cord injury in adult rats through neurotrophic and pro-angiogenic actions. PLoS One.

[20] Nauta AJ, Fibbe WE (2007). Immunomodulatory properties of mesenchymal stromal cells. Blood.

[21] Ishikane S, Ohnishi S, Yamahara K, Sada M, Harada K, Mishima K (2008). Allogeneic injection of fetal membrane-derived mesenchymal stem cells induces therapeutic angiogenesis in a rat model of hind limb ischemia. Stem Cells.

[22] Tambuyzer BR, Bergwerf I, De Vocht N, Reekmans K, Daans J, Jorens PG (2009). Allogeneic stromal cell implantation in brain tissue leads to robust microglial activation. Immunol Cell Biol.

[23] Le Blon D, Guglielmetti C, Hoornaert C, Quarta A, Daans J, Dooley D (2016). Intracerebral transplantation of interleukin 13-producing mesenchymal stem cells limits microgliosis, oligodendrocyte loss and demyelination in the cuprizone mouse model. J Neuroinflammation.

[24] Bergwerf I, De Vocht N, Tambuyzer B, Verschueren J, Reekmans K, Daans J (2009). Reporter gene-expressing bone marrow-derived stromal cells are immune-tolerated following implantation in the central nervous system of syngeneic immunocompetent mice. BMC Biotechnol.

[25] Le Blon D, Hoornaert C, Daans J, Santermans E, Hens N, Goossens H (2014). Distinct spatial distribution of microglia and macrophages following mesenchymal stem cell implantation in mouse brain. Immunol Cell Biol.

[26] Reekmans K, De Vocht N, Praet J, Le Blon D, Hoornaert C, Daans J (2013). Quantitative evaluation of stem cell grafting in the central nervous system of mice by in vivo bioluminescence imaging and postmortem multicolor histological analysis. Methods Mol Biol.

[27] De Vocht N, Reekmans K, Bergwerf I, Praet J, Hoornaert C, Le Blon D (2012). Multimodal imaging of stem cell implantation in the central nervous system of mice. J Vis Exp.

[28] Praet J, Santermans E, Reekmans K, de Vocht N, Le Blon D, Hoornaert C (2014). Histological characterization and quantification of cellular events following neural and fibroblast(-like) stem cell grafting in healthy and demyelinated CNS tissue. Methods Mol Biol.

[29] Bahmani P, Schellenberger E, Klohs J, Steinbrink J, Cordell R, Zille M (2011). Visualization of cell death in mice with focal cerebral ischemia using fluorescent annexin A5, propidium iodide, and TUNEL staining. J Cereb Blood Flow Metab.

[30] Adamczak JM, Schneider G, Nelles M, Que I, Suidgeest E, van der Weerd L (2014). In vivo bioluminescence imaging of vascular remodeling after stroke. Front Cell Neurosci.

[31] Chen J, Sanberg PR, Li Y, Wang L, Lu M, Willing AE (2001). Intravenous administration of human umbilical cord blood reduces behavioral deficits after stroke in rats. Stroke.

[32] Hamzei Taj S, Kho W, Aswendt M, Collmann FM, Green C, Adamczak J (2016). Dynamic modulation of microglia/macrophage polarization by miR-124 after focal cerebral ischemia. J NeuroImmune Pharmacol.

[33] Dooley D, Lemmens E, Vangansewinkel T, Le Blon D, Hoornaert C, Ponsaerts P (2016). Cell-based delivery of Interleukin-13 directs alternative activation of macrophages resulting in improved functional outcome after spinal cord injury. Stem Cell Rep.

[34] Ali I, Aertgeerts S, Le Blon D, Bertoglio D, Hoornaert C, Ponsaerts P (2017). Intracerebral delivery of the M2 polarizing cytokine interleukin 13 using mesenchymal stem cell implants in a model of temporal lobe epilepsy in mice. Epilepsia.

[35] Hoornaert CJ, Luyckx E, Reekmans K, Dhainaut M, Guglielmetti C, Le Blon D (2016). In vivo interleukin-13-primed macrophages contribute to reduced alloantigen-specific T cell activation and prolong immunological survival of allogeneic mesenchymal stem cell implants. Stem Cells.

[36] Tanaka R, Komine-Kobayashi M, Mochizuki H, Yamada M, Furuya T, Migita M (2003). Migration of enhanced green fluorescent protein expressing bone marrow-derived microglia/macrophage into the mouse brain following permanent focal ischemia. Neuroscience.

[37] Schilling M, Besselmann M, Leonhard C, Mueller M, Ringelstein EB, Kiefer R (2003). Microglial activation precedes and predominates over macrophage infiltration in transient focal cerebral ischemia: a study in green fluorescent protein transgenic bone marrow chimeric mice. Exp Neurol.

[38] Le Blon D, Hoornaert C, Detrez JR, Bevers S, Daans J, Goossens H, et al. Immune remodelling of stromal cell grafts in the central nervous system: therapeutic inflammation or (harmless) side-effect? J Tissue Eng Regen Med. 2016; 10.1002/term.2188. 10.1002/term.2188 27320821

[39] Garcia-Bonilla L, Faraco G, Moore J, Murphy M, Racchumi G, Srinivasan J (2016). Spatio-temporal profile, phenotypic diversity, and fate of recruited monocytes into the post-ischemic brain. J Neuroinflammation.

[40] Wattananit S, Tornero D, Graubardt N, Memanishvili T, Monni E, Tatarishvili J (2016). Monocyte-derived macrophages contribute to spontaneous long-term functional recovery after stroke in mice. J Neurosci.

[41] Evans TA, Barkauskas DS, Myers JT, Hare EG, You JQ, Ransohoff RM (2014). High-resolution intravital imaging reveals that blood-derived macrophages but not resident microglia facilitate secondary axonal dieback in traumatic spinal cord injury. Exp Neurol.

[42] Darsalia V, Kallur T, Kokaia Z (2007). Survival, migration and neuronal differentiation of human fetal striatal and cortical neural stem cells grafted in stroke-damaged rat striatum. Eur J Neurosci.

[43] Janata A, Magnet IA, Uray T, Stezoski JP, Janesko-Feldman K, Tisherman SA (2014). Regional TNFalpha mapping in the brain reveals the striatum as a neuroinflammatory target after ventricular fibrillation cardiac arrest in rats. Resuscitation.

[44] Shechter R, Miller O, Yovel G, Rosenzweig N, London A, Ruckh J (2013). Recruitment of beneficial M2 macrophages to injured spinal cord is orchestrated by remote brain choroid plexus. Immunity.

[45] Geissmann F, Jung S, Littman DR (2003). Blood monocytes consist of two principal subsets with distinct migratory properties. Immunity.

[46] Yamasaki R, Lu H, Butovsky O, Ohno N, Rietsch AM, Cialic R (2014). Differential roles of microglia and monocytes in the inflamed central nervous system. J Exp Med.

[47] Estevez AG, Sahawneh MA, Lange PS, Bae N, Egea M, Ratan RR (2006). Arginase 1 regulation of nitric oxide production is key to survival of trophic factor-deprived motor neurons. J Neurosci.

[48] Wang G, Zhang J, Hu X, Zhang L, Mao L, Jiang X (2013). Microglia/macrophage polarization dynamics in white matter after traumatic brain injury. J Cereb Blood Flow Metab.

[49] Hamzei Taj S, Kho W, Riou A, Wiedermann D, Hoehn M (2016). MiRNA-124 induces neuroprotection and functional improvement after focal cerebral ischemia. Biomaterials.

[50] Frank MG, Barrientos RM, Biedenkapp JC, Rudy JW, Watkins LR, Maier SF (2006). mRNA up-regulation of MHC II and pivotal pro-inflammatory genes in normal brain aging. Neurobiol Aging.

[51] Arnett HA, Wang Y, Matsushima GK, Suzuki K, Ting JP (2003). Functional genomic analysis of remyelination reveals importance of inflammation in oligodendrocyte regeneration. J Neurosci.

[52] Ohtaki H, Yin L, Nakamachi T, Dohi K, Kudo Y, Makino R (2004). Expression of tumor necrosis factor alpha in nerve fibers and oligodendrocytes after transient focal ischemia in mice. Neurosci Lett.

[53] Kuric E, Ruscher K (2014). Dynamics of major histocompatibility complex class II-positive cells in the postischemic brain—influence of levodopa treatment. J Neuroinflammation.

[54] Stellwagen D, Malenka RC (2006). Synaptic scaling mediated by glial TNF-alpha. Nature.

[55] Hanania R, Sun HS, Xu K, Pustylnik S, Jeganathan S, Harrison RE (2012). Classically activated macrophages use stable microtubules for matrix metalloproteinase-9 (MMP-9) secretion. J Biol Chem.

[56] Komohara Y, Ohnishi K, Kuratsu J, Takeya M (2008). Possible involvement of the M2 anti-inflammatory macrophage phenotype in growth of human gliomas. J Pathol.

[57] Yu B, Sondag GR, Malcuit C, Kim MH, Safadi FF (2016). Macrophage-associated osteoactivin/GPNMB mediates mesenchymal stem cell survival, proliferation, and migration via a CD44-dependent mechanism. J Cell Biochem.

[58] Xia W, Xie C, Jiang M, Hou M (2015). Improved survival of mesenchymal stem cells by macrophage migration inhibitory factor. Mol Cell Biochem.

