# Supplementary figures and images for: Targeted intracerebral delivery of the anti-inflammatory cytokine IL13 promotes alternative activation of both microglia and macrophages after stroke
Source: J Neuroinflammation. 2018 Jun 4;15:174. doi: 10.1186/s12974-018-1212-7 (PMC5987479; doi:10.1186/s12974-018-1212-7)

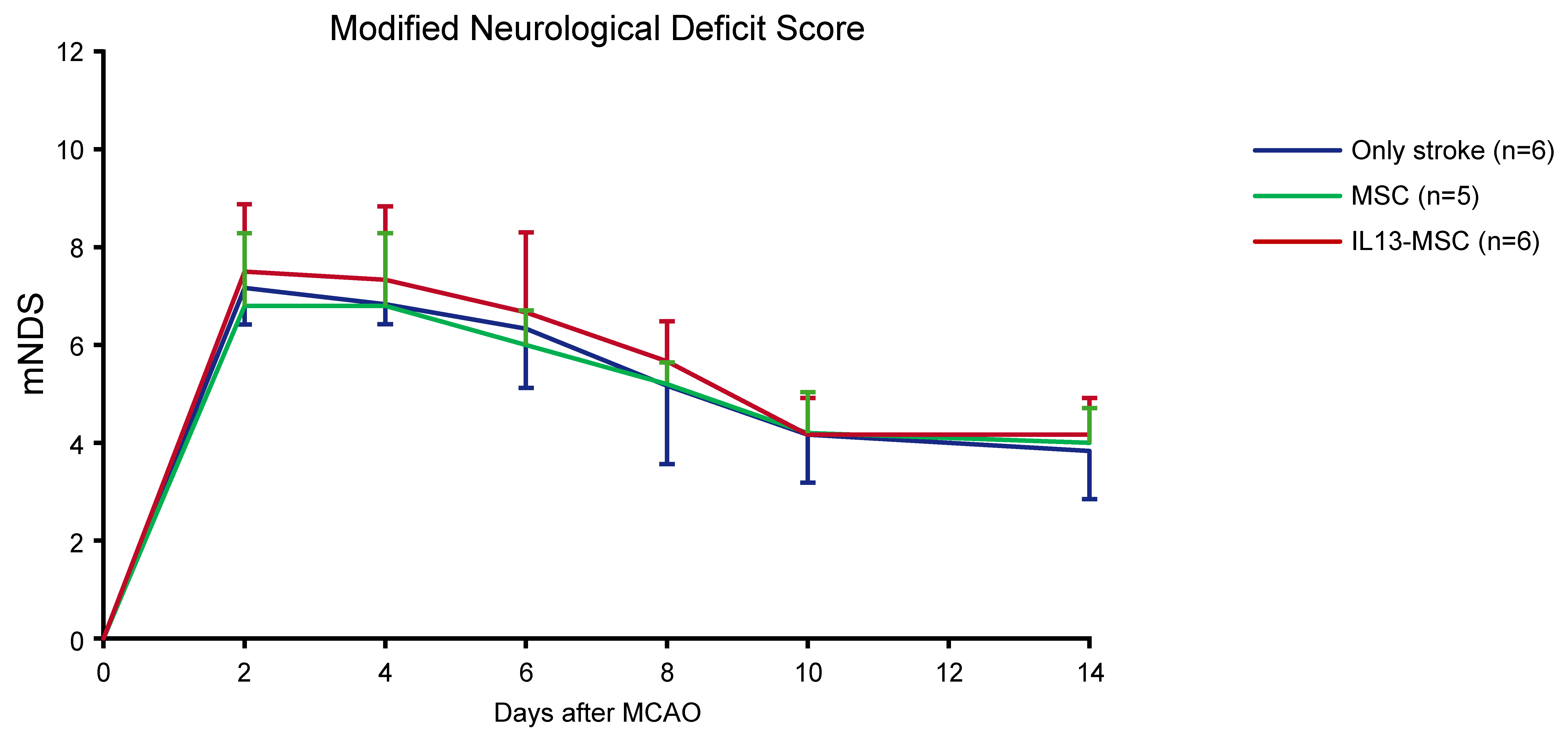

Supplement: Supplementary file 1 — Figure S1. Motor and sensory performance after ischemic stroke. Behavioral performance was assessed by the modified neurological deficit scores (mNDS), before and every 2 days after MCAO. In all three groups, the mNDS increased significantly at day 2 after MCAO compared to baseline. Graphs of all three experimental groups show no significant difference among the groups. The procedure of cell grafting into the ischemic brain and the additional surgical procedure did not worsen the motor and sensory performance after stroke. n = 5–6 mice in each group. Data are mean ± SD. The data were compared between the three experimental groups using the nonparametric Kruskal-Wallis H test. (TIF 837 kb) [file 12974_2018_1212_MOESM1_ESM.tif]

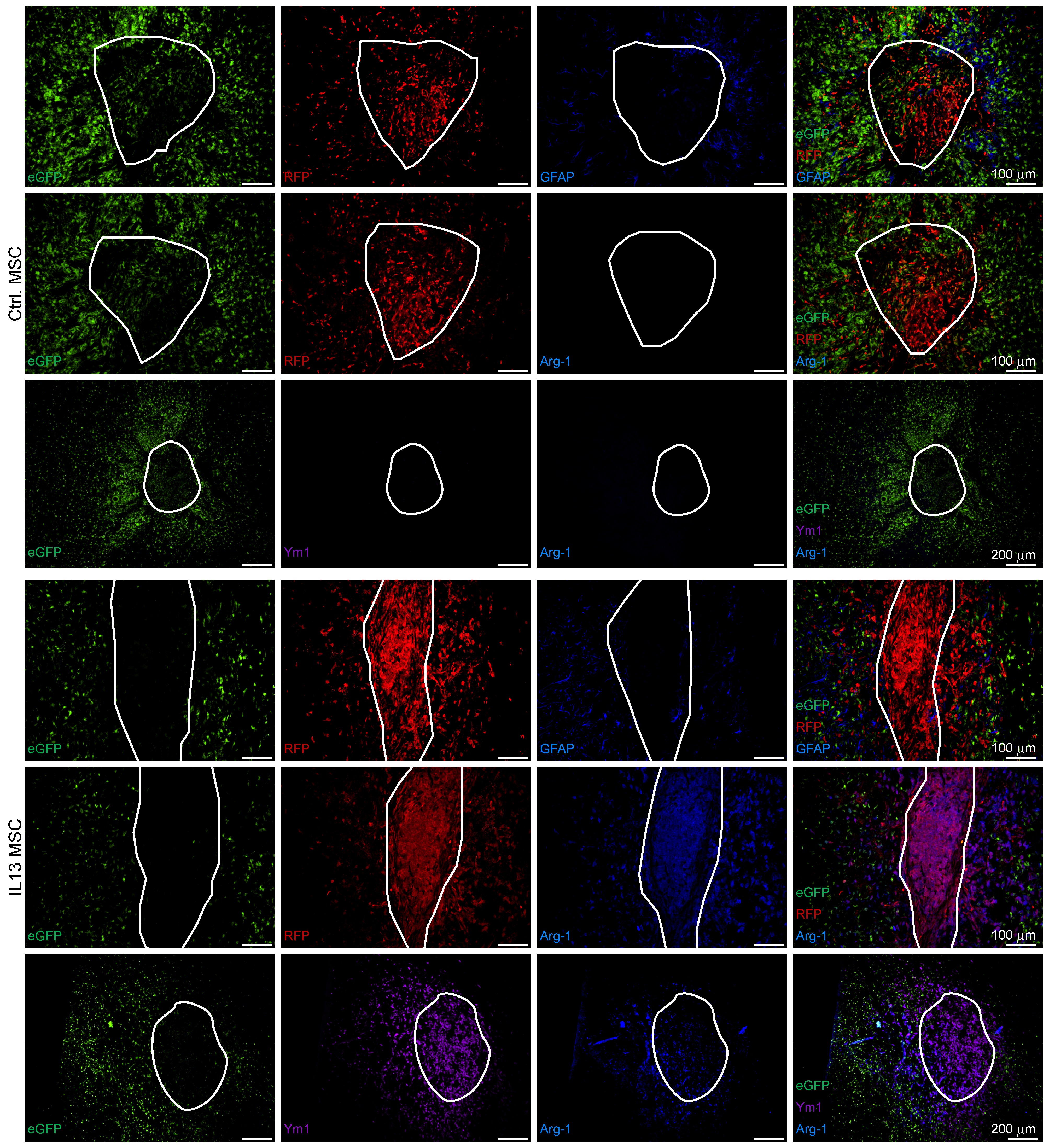

Supplement: Supplementary file 2 — Figure S2. Representative example of MSC and IL13-MSC graft site remodeling within the MCAO brain lesion site. Control MSC (upper panel) and IL13-MSC (lower panel) grafts are able to survive in the pro-inflammatory stroke environment and display a similar remodeling pattern. MSC graft-infiltrating CCR2RFP/+ monocytes/macrophages (in red) at the core of the MSC grafts and brain-resident CX3CR1eGFP/+ microglia (in green) and astrocytes (in blue, first row) surrounding the MSC grafts. Arginase1 expression (in blue, second and third row) and Ym1 expression (in magenta, third row) by microglia and monocytes/macrophages, as a direct result of stimulation by IL13, was only detected in IL13-MSC grafts, but not in control MSC grafts. Scale bar 100 μm. (TIF 13199 kb) [file 12974_2018_1212_MOESM2_ESM.tif]
